# A Survey on Hearing Health of Musicians in Professional and Amateur Orchestras

**DOI:** 10.1177/23312165241293762

**Published:** 2024-12-09

**Authors:** Robin Hake, Gunter Kreutz, Ulrike Frischen, Merle Schlender, Esther Rois-Merz, Markus Meis, Kirsten C. Wagener, Kai Siedenburg

**Affiliations:** 1 Department of Medical Physics and Acoustics, 11233 Carl von Ossietzky Universität Oldenburg, Oldenburg, Germany; 2 Institute of Music, 11233 Carl von Ossietzky Universität Oldenburg, Oldenburg, Germany; 3 9168 University of Bremen, Bremen, Germany; 4 Audienz – Musikalische Hörberatung e.U., Vienna, Austria; 5 167651 Cochlear Deutschland GmbH Co. KG, Hanover, Germany; 6 Hörzentrum Oldenburg gGmbH, Oldenburg, Germany; 7 Signal Processing and Speech Communication Laboratory, 27253 Graz University of Technology, Graz, Austria

**Keywords:** hearing health, noise exposure, hearing protection, hearing aids, musicians

## Abstract

Hearing health, a cornerstone for musical performance and appreciation, often stands at odds with the unique acoustical challenges that musicians face. Utilizing a cross-sectional design, this survey-based study presents an in-depth examination of self-rated hearing health and its contributing factors in 370 professional and 401 amateur musicians recruited from German-speaking orchestras. To probe the nuanced differences between these groups, a balanced subsample of 200 professionals and 200 amateurs was curated, matched based on age, gender, and instrument family. The findings revealed that two-thirds of respondents reported hearing-related issues, prevalent in both professional and amateur musicians and affecting music-related activities as well as social interactions. The comparative analysis indicates that professionals experienced nearly four times more lifetime music noise exposure compared to amateurs and faced more hearing challenges in social contexts, but not in musical settings. Professionals exhibited greater awareness about hearing health and were more proactive in using hearing protection devices compared to their amateur counterparts. Notably, only 9% of professional musicians’ playing hours and a mere 1% of amateurs’ playing hours were fully protected. However, with respect to their attitudes toward hearing aids, professional musicians exhibited a noticeable aversion. In general, an increase in music-related problems (alongside hearing difficulties in daily life) was associated with a decrease in mental health-related quality of life. This research highlights the importance of proactive hearing health measures among both professional and amateur musicians and underscores the need for targeted interventions that address musicians’ specific hearing health challenges and stigmatization concerns about hearing aids.

## Introduction

Playing musical instruments is associated with long-term health benefits (see the review by Dingle et al. (2021)). Yet, music-making, whether pursued at an amateur or professional level, may also pose inherent risks, including the potential for developing noise-induced hearing impairments (Musgrave, 2022; Schink et al., 2014). The likelihood of developing such impairments is influenced by the duration and intensity of noise exposure: both short but intense noise levels of 137 dB_peak_-C, as well as comparatively low but prolonged exposure at an average of 85 dB-A over several years, have been reported to result in permanent noise-induced hearing loss (HL) (Liedtke, 2010; “Königsteiner” and “NIOSH” recommendations; Chan, 1998; Deutsche Gesetzliche Unfallversicherung, 2013, 2020). Indeed, musicians regularly confront sound intensity levels that not only challenge but also endanger their hearing (O'Brien et al., 2013). Depending on their instrument, position, and repertoire, they often face peak sound levels of up to 135 dB-C and practice durations extending to 6 hours daily (Halevi-Katz et al., 2015; Rodrigues et al., 2014; Schmidt et al., 2011). Consequently, bound to their profession, musicians are inherently at risk for developing noise-induced hearing impairments (Gong et al., 2018; Schink et al., 2014).

A body of research highlights the risks musicians face in terms of hearing health. Phillips and Mace (2008) report that 48% of music students are exposed to harmful noise levels during daily practice. Similarly, O'Brien et al. (2013) found that 53% of musicians surpass the permissible daily noise exposure limits (see also Tufts & Skoe, 2018), a salient finding, as it does not include time spent in orchestral rehearsals and concert performances (i.e., only solitary practice was considered). It is therefore not surprising that Halevi-Katz et al. (2015) linked greater musical experience (years of persistent music playing) to higher hearing thresholds in the frequency range of 3–6 kHz. Furthermore, Hasson et al. (2009) reported that 40% of musicians experience some form of hearing disorder, with tinnitus (19%) being the most common, followed by 14% suffering from hyperacusis (one form of auditory hypersensitivity), and 6% experiencing difficulties in noisy environments or sound distortions—a finding that is consistent with Harper (2002), who recorded hearing problems among 38% of British and 37% of German musicians. Notably, a systematic review by Di Stadio et al. (2018) revealed that professional musicians in pop/rock genres are at an even higher risk of developing HL and other audiological symptoms compared to classical musicians. Gembris et al. (2018) further indicated that the likelihood of hearing problems of orchestra musicians escalates with age. The impact of HL extends beyond listening abilities, affecting mental health and social well-being. Bigelow et al. (2020) demonstrated that HL correlates with increased psychological distress, greater use of antidepressants, and higher dependency on mental health services among the general population.

However, the relationship between noise exposure and hearing impairment is not always straightforward. For example, Prendergast et al. (2017) had difficulties relating noise exposure to perceptual deficits in young listeners (mostly nonmusicians) with normal hearing (NH) audiometry. Couth et al. (2020) investigated this in a sample of musicians but could only find subclinical effects of noise exposure on hearing. Moreover, Russo et al. (2013) found no significant differences in hearing issues between instrument groups, even though brass, woodwinds, and percussionists have been reported to be exposed to particularly high sound levels (e.g., Rodrigues et al., 2014; Schmidt et al., 2011).

In summary, musicians commonly face hearing health challenges such as HL and tinnitus, impacting their musical performance and overall quality of life (Halevi-Katz et al., 2015; Hébert & Lupien, 2009; Richter, 2011). Furthermore, hearing health may directly and indirectly affect the ability to perceive, appreciate, and produce music, alongside impacting mental, physical, and social well-being (e.g., Bigelow et al., 2020; Gembris et al., 2018, Musgrave, 2022; Scherer, 2004, Zentner et al., 2008).

Recognizing the risks to hearing health in musicians, it becomes crucial to focus on their habits and preventive strategies. Certainly, adopting behaviors that reduce exposure to loud noise is essential for preventing hearing problems (e.g., Hébert & Lupien, 2009; Olsen et al., 2016; Richter, 2011). Intervention strategies for public health thus often include the promotion of hearing protection (e.g., Occupational Safety and Health Administration, 2002). Still, the actual usage of hearing protection among musicians and the barriers to its consistent application remain key concerns. Laitinen (2005), for example, found that among classical musicians in Finland, the use of hearing protection is sporadic, often commencing only after the onset of auditory symptoms. Specifically, a mere 6% consistently use hearing protection, while 35% seldom do—despite the fact that 31% reported some form of HL and 37% reported experiencing tinnitus. However, these are not uniformly distributed across regions. Among Australian musicians, 40% reported using hearing protection during practice, and 64% claimed occasional earplug use (O'Brien et al., 2013, 2014). This contrasts with orchestras in Germany, where only 38% reported occasional hearing protection use (Zander et al., 2008). Interestingly, there is some indication that younger musicians are more inclined toward protective measures: a study by Couth et al. (2021) found that among 80 British early-career musicians, 77% used hearing protection at least once a week.

The inconsistent use of hearing protection among musicians, despite the well-documented risks, demands an examination of the motivations behind these unhealthy choices. This exploration must consider the significant role of awareness in shaping health-related behavior. While awareness is often a precursor to behavioral change (e.g., Ajzen, 1991), one might assume that greater awareness of hearing health risks among musicians would naturally lead to increased use of hearing protection. For instance, studies by Keppler et al. (2015a, 2015b, 2015c) suggest that increasing awareness about hearing health substantially contributes to the adoption of protective measures against recreational noise. However, this inference is complicated by findings from Laitinen (2005), where a very high percentage (94%) of musicians reported concern for their auditory health, yet the actual use of hearing protection was notably low (6% consistent usage). This disparity suggests the presence of other barriers within the musical context, which may be intrinsic to the professional demands and social dynamics of musical performance. For example, musicians frequently cite issues with sound quality and difficulties hearing other musicians as significant obstacles to consistent hearing protection use (Laitinen, 2005; O'Brien et al., 2014; Zander et al., 2008). Additional factors such as varying loudness levels during performances or hampered self-monitoring could further discourage musicians from using hearing protection. Moreover, there is a less explored but equally compelling aspect that may hinder the consistent use of hearing protection among musicians—concerns about one's professional image. The use of hearing protection could potentially be perceived as a sign of auditory weakness, thereby affecting a musician’s reputation or audience perception (see Couth et al., 2021).

A similar dynamic may also contribute to the reluctance toward hearing aid adoption. Despite their proven utility in enhancing auditory experiences, hearing aids remain underutilized (e.g., Chern et al., 2023). While the literature does not specifically address the potential impact on musicians’ reputation as a factor in hearing aid adoption, musicians repeatedly complain that devices often are not effectively equipped or fitted to account for the full dynamics essential in music (e.g., Madsen & Moore, 2014). Recently, Greasley et al. (2020) extensively explored the complex dynamics of hearing aid usage among musicians by considering both audiologists’ and patients’ perspectives in the UK. Their study revealed another factor: a significant misalignment in expectations and understanding between these groups. Among musicians who use hearing aids, only 42% report an enhanced enjoyment of music while wearing them, with half experiencing distortions and over two-thirds encountering problems occasionally. A primary concern is the gap in audiologists’ training and understanding regarding the specific demands of hearing aids in musical environments. Only a small proportion (37%) of audiologists have received training focused on music, and despite 74% seeking additional information on this subject, many still express only moderate confidence in advising patients about music and hearing aids. Indeed, 58% of patients reported that music listening was never discussed. Consequently, many musicians feel their unique needs for high-quality music listening are not being met, leading to dissatisfaction and low adoption rates. These insights underscore a need for more specialized knowledge and tailor-made solutions in hearing aids for musicians. While awareness is a critical factor, it does not operate in isolation. The limited adoption of hearing protection and hearing aids is a complex interplay of personal, professional, and societal factors, warranting a multifaceted approach to intervention.

In sum, the literature suggests a link between musical noise and an increased risk of hearing problems in musicians, yet generalizing these findings across different groups and levels of exposure remains challenging. Existing studies predominantly focus on professional musicians, disregarding the large proportion of recreational (amateur) musicians—which amount to 14.3 million musicians in Germany alone (Deutsches Musikinformationszentrum, 2021). This necessitates a more detailed investigation into how music-related noise exposure affects musicians, particularly considering the varied intensity levels associated with different instrument groups, the range of environments in which music is performed (from individual practice at home to full orchestral performances), and the frequency and duration of musical activities (that is, considering both professional and amateur musicians). Despite this, the literature provides limited insight into the specific requirements necessary to implement preventive measures effectively. This gap extends to understanding how awareness and concerns about reputation influence musicians’ decisions, particularly regarding the use of hearing protection and the adoption of hearing aids.

### The Current Study

Addressing this gap, the current study aimed to provide a comprehensive investigation of hearing health among orchestral musicians, both professional and amateur. This included examining the utilization of and attitudes toward hearing protection and hearing aids, the prevalence and types of hearing problems, and their overall impact on musicians’ well-being. While the analysis was exploratory in nature, it was underpinned by two primary hypotheses: Professional musicians were expected to (a) report more hearing problems and (b) demonstrate a higher level of awareness toward hearing health compared to their amateur counterparts. Additionally, this research aimed to delve into the usage, experiences, and effectiveness of various strategies employed to protect hearing health. An important facet of this exploration was to identify and analyze the key factors that influenced the adoption rates of hearing protection and hearing aids among musicians. Subsidiary to these main goals, the study also explored the influence of demographic factors, specifically age as a critical factor in HL, on these relationships.

## Methods

### Participants

In total, 582 amateur musicians and 646 professional musicians initiated the survey. The recruitment strategy for this cross-sectional survey was multifaceted, targeting not only orchestral musicians but also choral musicians, conductors, and the orchestral management. However, this article focuses exclusively on orchestral musicians. Accordingly, nonorchestral musicians, participants who completed less than 50% of the survey, and those who took less than 7 minutes to complete the survey (which was identified as a minimal duration for any serious response strategy) were excluded from the analysis (*n* = 617). To further qualify musicianship among the amateur sample, a criterion based on the question “*How many years have you practised an instrument regularly and daily?*” was applied, using a cut-off of 6 years for regular practice (see Zhang & Schubert, 2019). This resulted in the exclusion of additional 16 participants.

#### Final Sample

After implementing the aforementioned exclusion criteria, the final sample comprised 282 professional musicians (*M*_age _= 49.2, SD = 11.3, range = 25–65 years; 130 females, one nonbinary, 12 not specified) and 313 amateurs (*M*_age _= 49.5, SD = 18.8, range = 25–85 years; 184 females, seven not specified). For a more nuanced description of the level of musical sophistication among the amateur musicians, see Figure A1 in Supplementary Materials.

### Procedure

The survey was administered from February 2022 to February 2023. Recruitment for this cross-sectional survey was conducted in collaboration with the German Orchestra Association through an internal newsletter, complemented by email invitations distributed to amateur orchestras within Germany and musicians in Austria. The questionnaire itself was provided by an online web application for creating online surveys called “SoSci Survey” (v.3.2.44; Leiner, 2019). Participants were explicitly asked to choose a survey version depending on whether they play music on an amateur or professional level. To accommodate linguistic diversity in orchestras, the questionnaire was available in both English and German, allowing for language selection at any point during the survey. Prior to commencing the survey, participants provided informed consent, acknowledging that participation was both anonymous and voluntary. Notably, no monetary or other incentives were offered for participation. All participants were instructed to complete the survey in their own time, and it was explicitly stated that participants could selectively skip questions if desired. On average, the survey took about 20 minutes to complete. This study was approved by the Carl von Ossietzky University Oldenburg ethics review board (Drs.EK/2021/114).

### Materials

Data were collected on demographic variables and musical background details, including age (categorized in 10-year intervals from “up to 30 years” to “80 + years”), gender, primary (contract) instrument, orchestra category, and repertoire. To assess HL, participants were asked to report their latest audiological reports if available. Those without prior audiological assessment completed an adapted version of the Hearing Aid for Music questionnaire (Greasley, 2022; see Table A1 and Table A2 in Supplementary Materials). HL was then categorized using the classification system of the World Health Organization (2001) and was then adapted from Martini (1996) to ensure the inclusion of milder forms of HL. This adaptation provides a more sensitive measure of HL, which is particularly important for assessing musicians who might experience subtle, yet impactful, changes in their hearing (see also Humes, 2019). Accordingly, in this study, the following adapted classification was used: mild HL: 20–40 dB HL (i.e., adapted from Martini, 1996), moderate: 41–60 dB HL (WHO), severe: 61–80 dB HL (WHO), profound: >81 dB HL (WHO).

Additional items targeted hearing protection usage. Specifically, participants were asked to report the number of hours they spend per week practising alone, rehearsing in groups, and performing at concerts—while also including measures on the frequency and type of hearing protection used during these activities. To further assess hearing health-related issues, the questionnaire incorporated several metrics: scales for estimating musical sound levels, measuring musical sophistication, evaluating hearing-related quality of life, scales to identify music-related and daily life hearing challenges, and a hearing aid disapproval scale.

#### Music Sound Level Exposure

The Noise Exposure Structured Interview (NESI; Guest et al., 2018) was adapted into a survey to measure musicians’ yearly noise exposure units (unit of noise exposure (UNE)). The UNE represents a typical yearly exposure to 90 dB-A noise over 2080 hours (52 weeks of 8-hour workdays). Accordingly, if a musician accrues one UNE over the period of one year, it is analogous to, for example, a construction worker, being exposed to 90 dB-A work-related noise for 52 weeks, 8 hours each working day. The UNE was calculated using the following equation:

$$UNE=\frac{W*44_{\left(weeks\right)}}{2080_{\left(hours\right)}}*\left(\;P*10^{\frac{L-A-90_{\left(dB\right)}}{10}}+(1-P)*10^{\frac{L-90_{\left(dB\right)}}{10}}\right)$$

where, *W* is the number of hours of instrumental noise exposure per week, 2080 corresponds to the number of hours in a working year, *P* is the proportion of time that hearing protection was worn (0 to 1), *L* denotes the noise level in dB-HL, and *A* represents the level of attenuation provided by hearing protection in dB. Hours of exposure were based on self-reported averages for practice, group rehearsals, and concert performances. Noise levels (L) were estimated from literature for different instruments and settings (see Tables A3–A4 in Supplementary Materials). Hearing protection usage frequency was converted to probability estimates (e.g., “always” = 100%). Attenuation values (A) were based on data from Guest et al. (2018; see Tables A5–A6 in Supplementary Materials). Lifetime music noise exposure (L-UNE) was derived by multiplying years of exposure by UNE and was log-transformed for analysis. Years of exposure for amateur musicians were calculated based on the reported years of playing an instrument. Notably, the exact years of exposure for professional musicians was not available. Instead, years of exposure were projected by assuming professional musicians would commence their musical education no later than the age of 10 years and begin to perform within orchestra rehearsals and concerts no later than the age of 20 years. This approach likely disregards a significant portion of years of instrumental noise exposure (average starting age of professional musicians have been reported to lie between 6 and 8 years, see Wesseldijk et al., 2020), yet also implements a rather conservative strategy in the estimation of lifetime music sound exposure. As participants reported both the type of hearing protection used and their usage frequency in various musical settings, UNE was calculated for individual practice, rehearsals and concerts separately and was then combined into a final aggregated score.

#### **Hearing-Dependent Daily Activities** (HDDA) **Scale**

The HDDA is a 12-item scale that assesses the impact of HL on daily life (Hidalgo et al., 2008). This scale includes subscales for the ability to perceive basic sounds in everyday life and a subscale assessing the impact of HL on social interactions. Participants responded to questions regarding their hearing abilities in daily life on a 3-point Likert scale (with the options “No, I can’t”; “With some difficulty”; “Yes, without difficulty”). The HDDA subscore for basic sound perception yields a final score ranging from 0 to 8, and social interactions spanning from 0 to 16. In both instances, a score of 0 denotes severely impaired hearing abilities. The HDDA is typically used for rapid HL screening, employing a cut-off score of >18 to indicate NH (Jacob et al., 2017). Here it is also used as a variable to estimate hearing difficulties in daily life settings.

#### **Goldsmith Musical Sophistication Index** (GMSI)

Amateur musicians were asked to complete the short version of the GMSI (Müllensiefen et al., 2014). The short GMSI is a brief, 18-item self-report questionnaire that assesses several aspects of musical expertise. It was designed to capture respondents’ overall level of musical sophistication by using subscales for active engagement, emotions, musical training, perceptual abilities, and singing abilities. Participants were asked to respond to a 7-point-Likert scale (1 =  completely disagree to 7 = completely agree). The final composite score ranges from 18 to 126, with higher scores indicating higher level of general musical sophistication.

#### **Health-Related Quality of Life** (VR-12)

Participant health-related quality of life was assessed through the application of the Veterans RAND 12 Item Health survey (Buchholz et al., 2017). This tool is widely recognized and validated for use across various demographic groups. The VR-12 questionnaire invites responses to statements such as “How would you describe your state of health in general,” which participants rate on a 5-point Likert scale (1 = “poor” and 5 = “excellent”). The survey culminates in two distinct subscales, the VR12-PCS and VR12-MCS, each of which are dedicated to the evaluation of physical and mental health, respectively. Both subscales are scored within a range of 0 to 100, with higher scores indicative of a higher reported quality of life.

#### Music-Related Hearing Problems (MRHP) Scale

Participants rated six items (e.g., “I have the impression that the sound is washed out/ blurred”) on a 5-Point-Likert-Scale, ranging from 1 = “always” to 5 = “never.” The latent one-factor structure was confirmed through confirmatory factor analysis (CFA), showing an internal consistency of α = .82 and R^2 ^= .44. Final scores on the scale span from 0 to 10, where higher values are indicative of more severe MRHP (see Table A7 in Supplementary Materials for details).

#### Hearing Health Awareness (HHA) Scale

The HHA scale is composed of seven items (e.g., “*Hearing health is extremely important to me.”* on a 4-Point-Likert-Scale; 1 = “true/applies” to 4 = “not true/does not apply.” The scale encompasses aspects such as the perceived strength of sound level exposure while playing music (n = 3), general attitudes toward hearing health (n = 3), and the attitude about wearing hearing protection while performing music (*n* = 1). The CFA achieved a good model fit with an *R*^2^ value of .33 and a Cronbach's alpha of .74. The final scores span from 0 to 10, with higher values being indicative of higher HHA (see Table A8 in Supplementary Materials for details).

#### Hearing Aids Disapproval (HAD) Scale

The HAD utilized five items (e.g., “*It irritates me, when colleagues with hearing impairments wear hearing aids.*”), encompassing aspects such as underlying apprehensions, societal perceptions, and potential stigma associated with the use of hearing aids among musicians (4-point Likert scale, 1 = “true/applies” to 4 = “not true / does not apply”). The CFA yields an internal consistency of *α* = .79 with an *R*^2 ^= .44. The scores derived from this scale range from 0 to 10, where higher values indicate a greater disapproval toward hearing aids (see Table A9 in Supplementary Materials for details).

### Analysis

All statistical analyses were conducted using R statistical software (version 2022.07.2 + 576; RStudio, 2020). Given the non-normally distributed nature of the data set, the Wilcoxon rank-sum test (also known as the Mann–Whitney *U* test, abbreviated as *W* [sample *n*]) was utilized for comparing two groups. For comparisons involving more than two groups, the Kruskal–Wallis test was employed, abbreviated as *H* [degree of freedom 1, degree of freedom 2]. Post-hoc comparisons involved pairwise Wilcoxon rank-sum tests with a Bonferroni correction to control Type I error rates. Effect sizes and confidence intervals, in addition to *p*-values, were reported to provide a more comprehensive understanding of the results. The rank-biserial correlation (*r*_rb_; see Kerby, 2014) was used as the measure of effect size for the Wilcoxon rank-sum tests, with *r*_rb_ values ranging from 0 to |1|. Values approaching 0 indicate a negligible effect, while an *r*_rb_ greater than .1 suggests a small effect. An *r*_rb_ exceeding .3 is indicative of a medium effect, and those above .5 signify a large effect. To supplement the Kruskal–Wallis test results, the Epsilon square measure (*ε*^2^) was reported to quantify effect size. Epsilon square values also range from 0 to 1, with higher values indicating stronger effects. The median (Mdn) and the median absolute deviation (MAD) were reported to address the nonparametric nature of the data. MAD, as a measure of statistical dispersion, indicates variability, with higher MAD values suggesting greater variability. Spearman’s rank-order correlation was used to assess relationships between continuous and ordinal data. The Chi-squared test was applied for categorical variables. In cases of missing data, listwise deletion was employed, leading to varying sample sizes for each analysis. The investigation centers on a global perspective on the data by mainly focusing on the results of the developed scales which summarize multiple items of the survey. To establish reliable scales for three latent constructs, the CFA was applied via the *lavaan* package (v06-15; Rosseel, 2012) in R (RStudio, 2020), utilizing all the data prior to any exclusions. The internal consistency of the scale was assessed by calculating Cronbach’s alpha, and various model fit indices and percentage of explained variances were reported to ensure the robustness of the established scales. For all three scales, weighted least squares mean and variance adjusted (WLSMV) estimator was used. Additionally, the interested reader may access the detailed data of every individual item in the corresponding open access dataset (see Hake et al., 2024).

## Results

### Musician Group Matching

**Figure 1.**
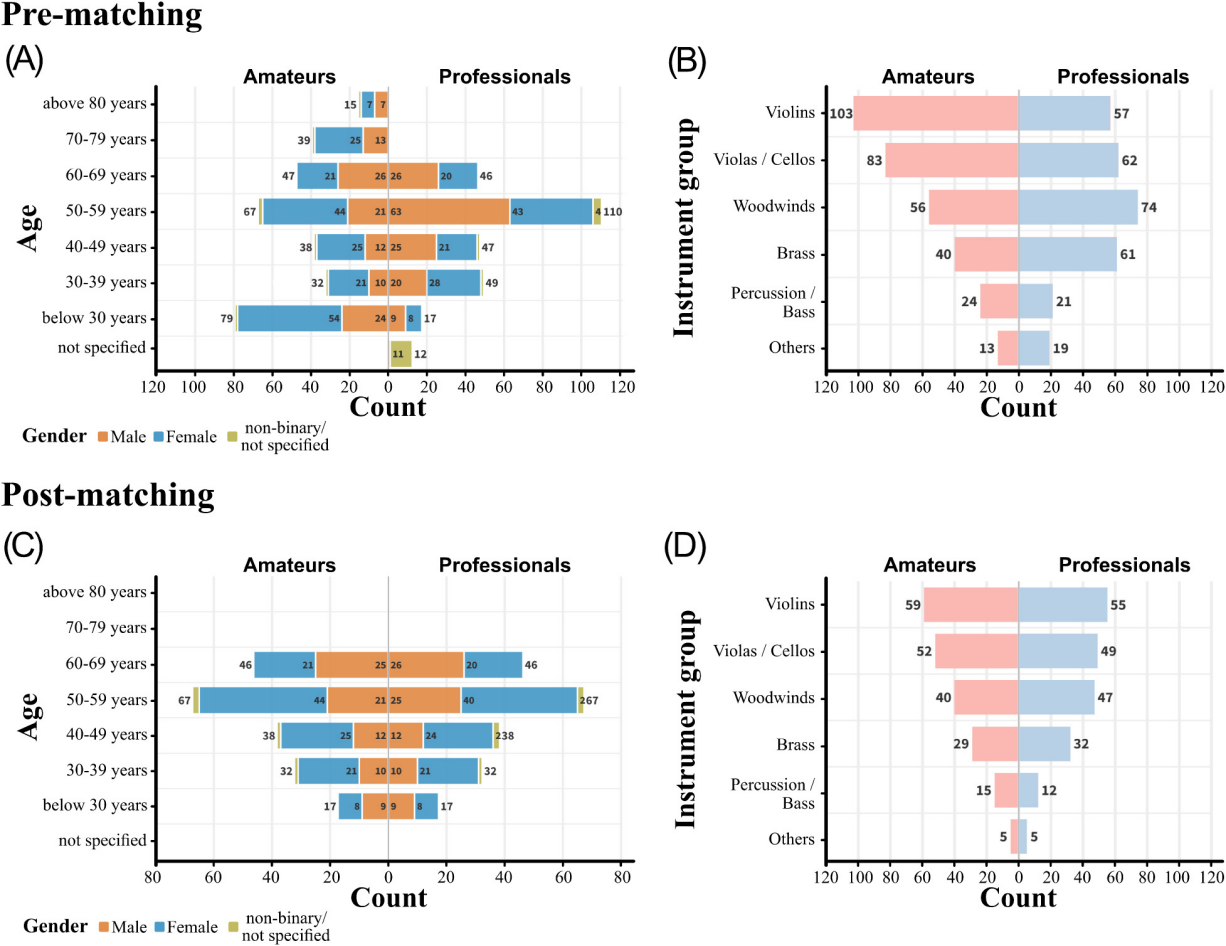
Demographic profiles of amateur (pale red) and professional musicians (light blue) as a function of age, gender (A, C), and instrument group distributions (B, D) before (upper row) and after the matching procedure (lower row).

Unmatched groups in comparative analyses can introduce significant bias due to confounding variables, thereby undermining the internal validity of the analysis. To probe for preexisting differences between professional and amateur musicians, a Chi-square test of independence was conducted, showing an significant association between group membership (amateur vs. professional) for age, *χ*²(7) = 133.6, *p* < .001. Upon further inspection it became apparent that comparably few young (up to 30 years) and senior professional musicians (70 years and above) participated in the survey compared to the amateurs (see Figure 1A). Similarly, there were significant differences between group membership for gender, *χ*²(3) = 8.3, *p* = .004, and for participants instrument family, χ²(5) = 23.5, *p* < .001 (see Table A3 in Supplementary Materials for the grouping of instruments). Thus, participants’ musical group membership (professionals vs. amateurs) demonstrated imbalance across age, gender, and instruments family.

In order to mitigate confounding effects, a matching algorithm was applied, utilizing the *MatchIt* package (v4.5.3; Ho et al., 2007). This package serves as a statistical tool designed to ensure comparability between professional musicians and amateurs by balancing the participant groups on several key demographics: age, gender, and instrument family of their primary (contract) instrument. A combination of the “optimal” and “exact” matching procedures was applied. The most critical aspect of this approach was the exact matching on age, since it significantly influences hearing. This method ensured that each pair of participants—comprising one professional and one amateur musician—was matched “exactly” within the same age group. For the variables of gender and instrument family, the “optimal” matching method was selected, coupled with the “Euclidean” distance metric. Rather than matching each pair precisely, this strategy aimed to minimize the overall distance across pairs in these two dimensions. Essentially, the result is a subsample in which each professional musician was balanced strictly in terms of age, with a more flexible distribution for gender and instrument family. This method allowed for a more nuanced matching, taking into account the combined effects of age, gender, and instrument family, while ensuring that a sufficient number of participants are retained for subsequent analysis.

After matching, 82 unmatched subjects from the professional musician group, and 113 from the amateur group were excluded. Accordingly, a total of 400 participants remained for the analysis. The distributions for age, gender, and instrument group before and after matching are represented in Figure 1. In conclusion, the matching process successfully balanced the key variables of age, gender, and instrument family between the professional and amateur musicians. That is, there are no statistically significant differences between groups neither in age, *χ*²(4) = 0, *p* = 1, gender, *χ*²(3) = 1.3, *p* = .73, and instrument group, *χ*²(5) = 1.27, *p* = .94, after matching. It is critical to note that despite the improvements in balance across these variables, other imbalances remained post-matching, for example, in repertoire played by the orchestra (see Figure A2 in Supplementary Materials). The demographic distribution of the final sample closely mirrors that of professional musicians in Germany, with deviations from the reference populations remaining within a 5% proportional margin. Thus, the sample presented here can be regarded as representative (see Versorgungsanstalt der deutschen Kulturorchester, 2021).

### Hearing-Related Problems

Participants who had received professional audiometric evaluations were instructed to report the final results of those assessments, specifically indicating whether they had been diagnosed with mild, moderate, severe, or profound HL. For participants who had not undergone professional audiometric assessments in the past and were thus unaware of their specific degree of HL, they were instructed to self-estimate their HL. Almost all professional musicians stated to have their hearing checked (97%), whereas only 72% of amateurs had their hearing checked by a professional at least once before. On average, the last appointment was 2.7 years ago (SD = 3.8) for professionals and 5.2 years (SD = 6) for amateurs.

**Figure 2.**
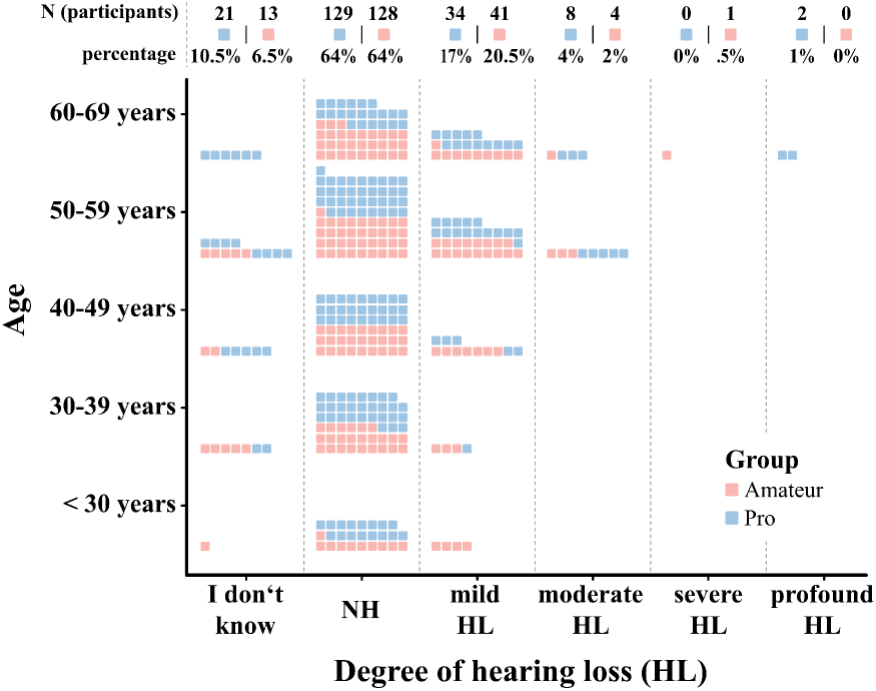
Distribution of reported HL among amateurs (pale red) and professional musicians (light blue) by age group. Both the self-reported audiological diagnoses and self-estimated HL are considered. Accordingly, 5 responses from professionals and 56 from amateurs are based on self-estimated HL rather than self-reported diagnosis from professional hearing screening. Note: *NA* values have been omitted, so percentages do not accumulate to 100%.

Both self-estimated and self-reported results of professionally diagnosed HL were included in the analysis. Overall, 22% of professionals (*N* = 44) and 23% of amateurs (*N* = 46) reported having at least a mild form of HL (see Figure 2). In addition, 24% of musicians (23.3% amateurs, 24.7% professionals) reported having tinnitus, 49.1% (44.8% amateurs, 53.3% professionals) reported being oversensitive to high sound levels, and 15.9% (11.9% amateurs, 18% professionals) reported other hearing problems. There were no significant differences of reported hearing problems across groups (professionals vs. amateurs) for the degree of HL, χ²(1) = 4.1, *p* = .26, tinnitus, *χ*²(1) = 1.84, *p* = .22, oversensitivity, *χ*²(1) = 2.87, *p* = .1, or reporting other problems, χ²(1) = 2.8, *p* = .12. Taken together, two-thirds (66.75%) of the respondents reported experiencing at least one of these kinds of hearing problems—a pattern that is indifferent to the musician’s group membership, with 67.5% of amateurs and 66% of professionals affected. Similarly, employing the HDDA HL screening cutoff (<18 indicating mild impairment; Jacob et al., 2017), a total of 107 individuals (48 amateurs, 59 professionals) were classified as hearing impaired. A chi-squared test found no significant difference in this classification between the groups, *χ*²(1, *N* = 385) = 1.37, *p* = .242, further suggesting that the proportion of individuals with hearing difficulties may be similar across both groups.

**Figure 3.**
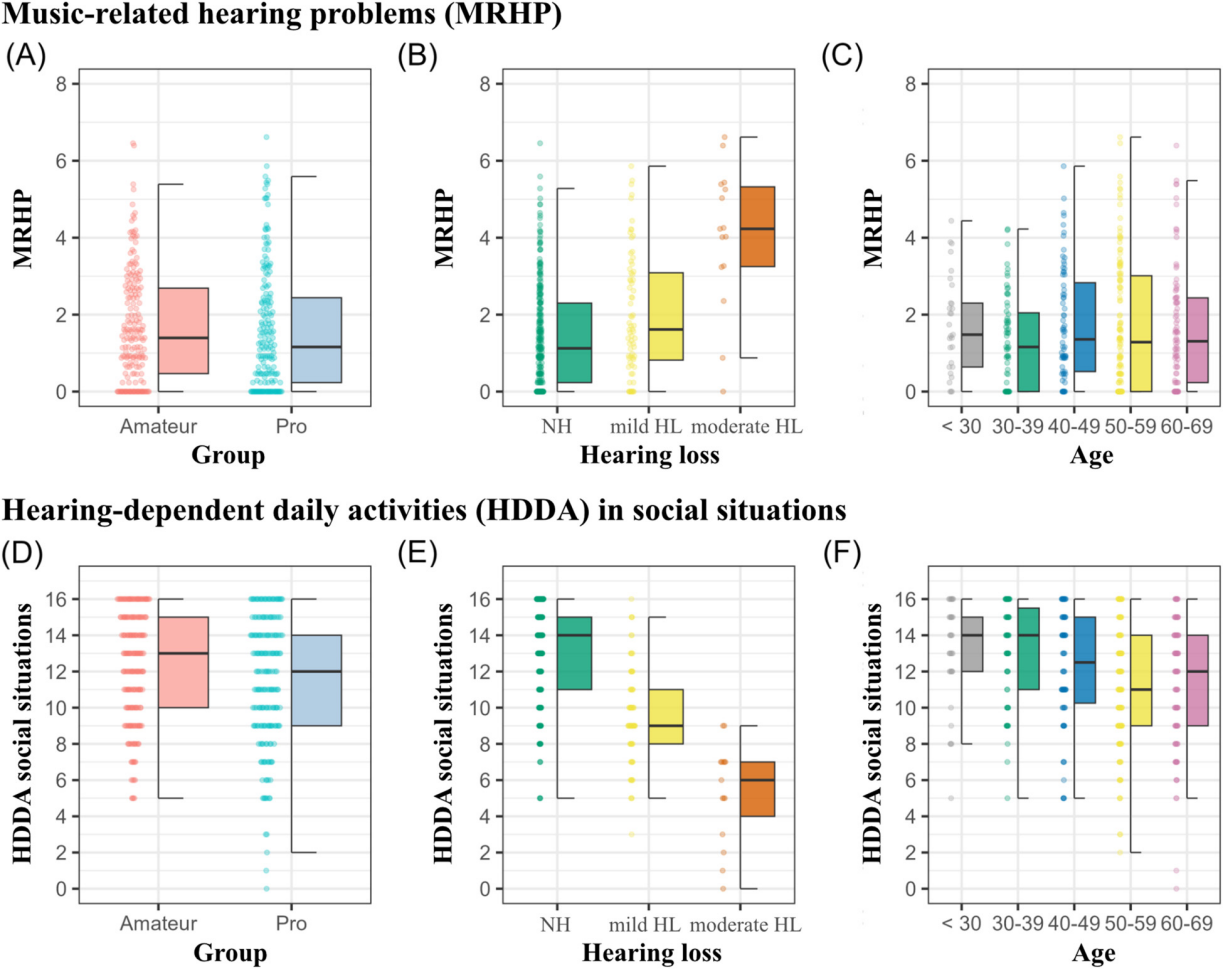
Amateur (pale red) and professional musicians (light blue) scores for the MRHP scale (higher scores equals more severe problems) and the HDDA (lower scores equals more severe problems). Panel A and D compare MRHP and HDDA in social situations scores between amateur and professional musicians. Panels B and E categorize MRHP and HDDA in social interactions by HL severity (NH, mild HL, at least moderate HL). Panels C and F illustrate MRHP and HDDA in social interactions across age cohorts. Here and in the following, boxplots represent the median and interquartile ranges of scores on the right side and individual responses on the left and *NA* values are excluded.

Furthermore, the analysis did not suggest any significant difference in the MRHP scale between the groups of musicians, Mdn_diff. _= .24; *W* = 18554, *p* = .3, *r*_rb_ (rank biserial) = .06, 95% CI [−.06, .18]; see Figure 3. Differences were observed, however, in the HDDA scale between the musician groups. Professional musicians reported more problems in social interaction (Mdn = 12, MAD = 4.45), compared to amateur musicians (Mdn = 13, MAD = 2.97). Lower scores indicate more severe hearing problems. The Wilcoxon rank-sum test revealed this difference to be statistically significant, *W*(391) =  21,519, *p* = .03. The effect size was *r*_rb _= .13, indicating a negligible effect. There were no statistically significant differences between the groups in the HDDA subscale for basic sound perception, *W*(391) =  14,300, *p* = .59.

#### Influence of HL and Age

When conducted irrespective of musicians’ group membership (also excluding “I don’t know” responses), a Kruskal–Wallis test indicated statistically significant differences in MRHP scores across the participants’ degree in hearing-impairment, *H*(2, 374) = 27.4, *p* < .001, with a subtle effect size (*ε*² = .07; see Figure 3). The median MRHP factor score was 1.14 (MAD = 1.7) for musicians with NH, 1.61 (MAD = 1.8) for the “mild hearing loss” (mild HL), and 4.23 (MAD = 1.5) for the “at least moderate HL” (moderate HL). A post-hoc pairwise Wilcoxon rank-sum test revealed that the differences were significant between the “NH” and the “mild HL” (*p* = .02), between the “NH” and the “at least moderate HL” (*p* < .001), as well as between the “mild HL” and the “at least moderate HL” group (*p* = .001). Likewise, HDDA scores showed significant effects with the reported degree of HL. The Kruskal–Wallis test revealed significant variance across the HL types, *H*(2, 391) = 100.4, *p* < .001, with a moderate effect size (*ε*² = .27). The Wilcoxon post-hoc test confirmed significant differences between all groups, that is, “NH” (Mdn =14, MAD = 3) and both “the mild HL” (Mdn = 9, MAD = 3) and “at least moderate HL” (Mdn = 6, MAD = 1.5), and also between the latter two (all comparisons reach *p* < .001).

No statistically significant differences were found for participants’ MRHP scores between age groups, *H*(4, 359) = 3.23, *p* = .52. In contrast, significant age-related differences were observed in the HDDA subscore for social interactions, *H*(4, 400) = 23.2, *p* < .001, although the overall effect size was marginal (*ε*² = .06). Post-hoc analysis revealed statistically significant differences between the “below <30” (Mdn = 14, MAD = 3) and “50–59” age groups (Mdn = 11, MAD = 3; *p* = .03), as well as between the “30–39” (Mdn = 14, MAD = 3) and both the “50–59” (*p* = .001) and “60–69” (Mdn = 12, MAD = 4.5, *p* = .007) age groups. No other age group comparisons yielded statistically significant results.

### Use of Hearing Protection

Overall, a majority of participants (59.4%, *N* = 240) acknowledged that playing music poses a risk to their hearing health due to the exposure to high sound levels; however, merely 52% of those who recognized the risk chose to wear any hearing protection. More strikingly, over 35% of respondents who affirmed the statement (respond with “true”)—“*The sound levels in the orchestra are so high at times that I should wear ear protection*”—and 77% of those who concurred with “rather true”, abstained from using hearing protection all together. In group comparisons, professionals exhibit a markedly higher tendency to use hearing protection compared to their amateur counterparts (53.5% of professionals vs. 12% of amateurs). However, the majority of musicians reported rarely or never using hearing protection, with percentages ranging from 72.5% to 82.4% for amateurs and from 14.5% to 70.6% for professionals, depending on the playing context (Detailed statistics are provided in Table 1). Notably, only 4% of professional musicians reported always using hearing protection during individual practice, compared to .5% of amateurs. This discrepancy widens during rehearsals, with 9.9% of professionals versus 0% of amateurs always using hearing protection. The trend persists during concerts, where 10.4% of professionals and only 1.5% of amateurs consistently use hearing protection. By combining reported hearing protection usage with individuals’ total instrumental playing time (similar to the method used for estimating the probability of attenuation in the UNE), it was found that amateurs spent 79.5% of their playing time unprotected, 19.5% partially protected (e.g., using protection in one ear or occasionally removing it), and 1% fully protected. In contrast, professional musicians spent 34% of their playing time unprotected, 57% partially protected, and 9% fully protected.

**Table 1.** Descriptive Statistics of Hearing Protection Usage by Professional and Amateur Musicians.

Use of hearing protection	Never	Rarely	Sometimes	Mostly	Always	Not applicable
Individual practice time						
Amateur	160 (82.4%)	15 (7.8%)	7 (3.6%)	3 (1.5%)	1 (.5%)	8 (4%)
Professionals	137 (70.6%)	27 (13.9%)	13 (3.7%)	4 (2%)	8 (4%)	5 (2.6%)
During rehearsals						
Amateur	140 (72.5%)	17 (8.8%)	22 (11.4%)	7 (3.6%)	0	7 (3.6%)
Professionals	28 (14.5%)	62 (32.3%)	59 (30.7%)	22 (11.5%)	19 (9.9%)	2 (1%)
During concerts						
Amateur	148 (77.1%)	20 (10.4%)	9 (4.7%)	3 (1.5%)	3 (1.5%)	9 (4.7%)
Professionals	58 (30.2%)	46 (24%)	52 (27%)	14 (7.3%)	20 (10.4%)	2 (1%)

*Note. N* = 400; “NA” values are omitted.

When examining the consistent use of hearing protection in conjunction with the degree of HL, a significant trend was observed, *H*(2, 364) = 13.25, *p* = .001. Specifically, analyzed irrespective of musicians group membership, 3% (SD = .15) of respondents with NH always used hearing protection. This percentage increased to 5% (SD = .19) among those with mild HL and surged to 22% (SD = .36) among individuals with at least moderate HL. Post-hoc pairwise Wilcoxon rank-sum tests revealed significant differences between those with at least moderate HL and mild HL (*p* < .001), as well as between those with at least moderate HL and NH (*p* = .03).

### Noise Exposure

Professional musicians reported significantly higher practice and performance durations than their amateur counterparts. On average, professionals engaged in 7.1 hours (SD = 4.2) of weekly practice for concerts, whereas amateurs reported an average of 8 hours (SD = 1.4). Rehearsal times followed a similar pattern, with professionals practising 15.2 hours weekly (SD = 5.9), in contrast to 3 hours (SD = 2.3) for amateurs. Besides group performances, professionals also practised more (i.e., individual practice), averaging 8.6 hours per week (SD = 5.1) compared to amateurs’ 3.6 hours (SD = 3.7).

**Figure 4.**
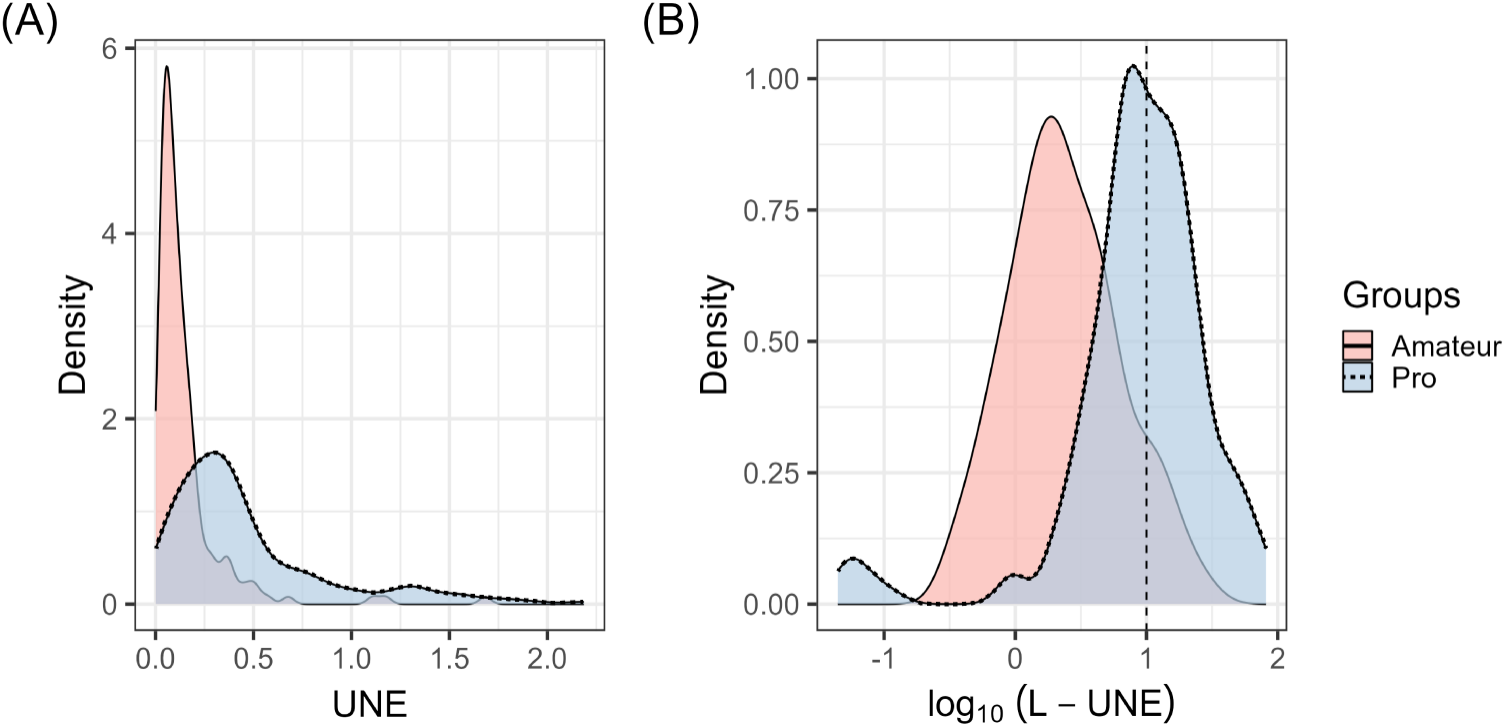
Musical sound exposure per year (UNE) and the cumulative (lifetime) music sound exposure (L-UNE) for amateur (pale red) and professional musicians (light blue).’Density’ refers to the empirically estimated probability density function.

Based on these practice habits, instrument groups, and hearing protection usage, participants’ self-reported musical UNE was derived. Following the classification by Prendergast et al. (2017), individuals with a log_10_(L-UNE) of one or higher were categorized as having high noise exposure—equivalent to a decade of exposure at 90 dB-A. According to this criterion, a proportion of 50% of professional musicians were classified within the high noise exposure group, a figure that contrasts with the 9.9% of amateur musicians, as illustrated in Figure 4.

In addition, the Wilcoxon rank-sum test revealed significant group differences in UNE and L-UNE: Professional musicians registered almost three times higher median levels of musical sound exposure (UNE_Mdn _= .34, MAD = .25) and almost four times higher lifetime music noise exposure (L-UNE_Mdn _= 10, MAD = 8.1), relative to their amateur counterparts, who reported lower levels of UNE_Mdn _= .09 (MAD = .07) and L-UNE_Mdn _= 2.1 (MAD = 1.91). These differences were statistically significant for both UNE, *W*(364) = 5828, *p* < .001, and L-UNE, W(364) = 5291, *p* < .001. The effect sizes were considerable, with *r*_rb _= −.65, 95% CI [−.71, −.57] for UNE and *r*_rb _= −.68, 95% CI [−.74, −.61] for L-UNE. Notably, 57% of amateurs’ lifetime music noise exposure have been experienced during individual practice, 33% during rehearsals, and 10% during concerts. In contrast, professionals experienced 49.4% during practice, 34.2% during rehearsals, and 16.4% during concerts.

#### Influence of HL and Age

In examining the association between UNE and age, the following pattern emerged: UNE was found to be highest among the youngest participants, with those under 30 years showing a median UNE score of .35 (MAD = .34). Overall, the UNE score decreased with age, reaching its lowest values in the 40–49 age group, with a median score of .14 (MAD = .16), and remaining that low for the rest of the musician’s career (see Figure A3 in Supplementary Materials). This effect was statistically significant, *H*(2, 364) = 23.2, *p* < .001, demonstrating a marginal effect size (ε² = .04). Post-hoc analyses utilizing pairwise *t*-tests revealed that the “below 30” age group is significantly higher when compared with the “40–49” (*p* < .01), the “50–59” (Mdn = .16, MAD = .17, *p* < .01), and “60–69” (Mdn = .18, MAD = .19, *p* = .04) age groups. With respect to the degree of HL, no significant differences were observed—neither for the UNE, *H*(2, 364) = .58, *p* = .75, nor the L-UNE, *H*(2, 364) = 1.02, *p* = .6.

### Hearing Health Awareness

**Figure 5.**
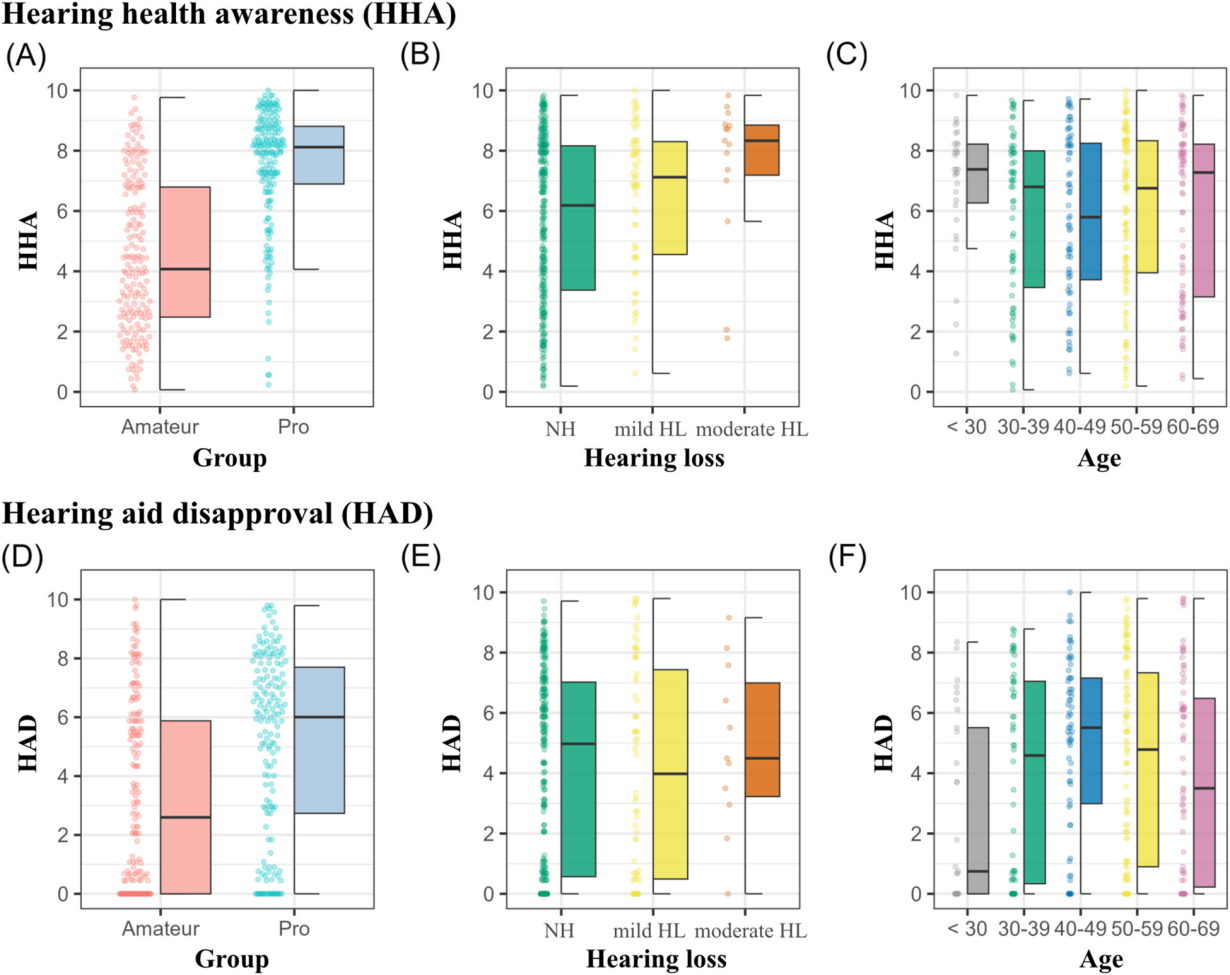
Scores of for the scales HHA (top row) and HAD (bottom row) as a function of group (panels A, D), HL (B, E), and age (C, F).

In the present study, musicians generally perceived hearing health as a topic that does not receive enough discussion, with 71.5% affirming this view as either “rather true” or “true”. This sentiment was slightly more common among amateurs (78.5%) compared to professionals (64%). Furthermore, a significant majority (74.75%) expressed a desire for regular counselling on the topic of hearing health and hearing impairment (at least “rather true”), with a higher interest demonstrated by professional musicians (81.5%) compared to amateurs (68%). However, a quarter of respondents (24%) expressed reluctance, responding with “not true” or “rather not true” when asked if they would discuss their HL openly. These rather negative attitudes toward hearing health were further reflected in the HHA factor scores. Overall, amateur musicians exhibited lower average awareness scores (HHA_Mdn _= 4.1, MAD = 3) compared to professional musicians (HHA_Mdn _= 8.1, MAD = 1.3). This difference was statistically significant, *W*(359) = 5711, *p* < .001, with a very strong effect size (*r*_rb _= −.65, 95% CI [−.71, −.6]), suggesting a substantial difference in HHA between amateur and professional musicians (see also Figure 5).

#### Influence of HL and Age

The HHA factor scores also revealed significant differences across HL categories, *H*(2, 359) = 9.4, *p* = .01; see Figure 5B. The NH group had a median HHA score of 6.2 (MAD = 3.3), the “mild HL” group 7.1 (MAD = 2.4), and the “at least moderate HL” group 8.3 (MAD = 1.4). However, the effect size was rather small (ε² = .03). The corresponding post hoc test found only the difference between the “NH” and “at least moderate HL” group in HHA scores to be statistically significant (*p* = .03). In terms of age, no differences in HHA factor scores could be observed, *H*(4, 359) = 3.2, *p* = .52.

### Hearing aid Disapproval

The analysis of the HAD scale revealed significant differences in approval ratings between amateur and professional musicians. Professional musicians exhibited higher levels of disapproval toward hearing aids (HAD_Mdn _= 6, MAD = 3.2) compared to their amateur counterparts (HAD_Mdn _= 2.6, MAD = 3.9; see Figure 5). This difference was statistically significant, *W*(335) = 9584.5, *p* < .001, and demonstrated a moderate effect size, *r*_rb _= −.32, 95% CI [−.42, −.2].

#### Influence of HL and Age

Moreover, the data indicated a relationship between age and HAD scores, with the Kruskal–Wallis test showing statistically significant differences and a small effect size, *H*(4, 335) = 11.44, *p* = .02, ε² = .03. Pairwise post-hoc tests indicated that the youngest age group “below 30 years” exhibited lower disapproval scores (Mdn = .75, MAD = 1.1) compared to the 40–49 years age group (Mdn = 5.51, MAD = 2.8), which showed the highest disapproval ratings, *p* = .015 (see Figure 5F). Despite these differences, an overall trend related to age was not observed, as Spearman's correlation revealed a nonsignificant relationship between participants’ age and their HAD scores, *r*(333) = .05, *p* = .396. Conversely, HAD scores did not significantly vary across musicians with different types of HL, *H*(2, 335) = .44, *p* = .8; see Figure 5E.

### Hearing aid Usage

The proportion of respondents using hearing aids was low. Only 7.8% of respondents (*N*_total _= 31) reported using hearing aids or having tried them in the past. This usage rate was slightly lower among amateurs (6.5%, *N* = 13) compared to professionals (9%, *N* = 18). The average duration of hearing aid use among these individuals was approximately 9 years, with a slightly longer average usage reported among amateurs (9.8 years) versus professionals (8.6 years). Among those participants who reported wearing hearing aids, a large majority (79.7%) reported improvements in their hearing (“agree” or “totally agree”). However, the benefits from hearing aids appeared to be uneven across different auditory tasks. Those respondents who reported to use hearing aids regularly (*N*_total _= 15) found hearing aids to be at least somewhat helpful for speech (100%). Similarly, 73% (*N* = 11) reported benefits for listening with ambient noise in the background. However, the effectiveness of hearing aids for music listening was deemed inadequate by many, with only 60% (*N* = 9) finding them useful for this purpose. This issue was further underscored by 54% (*N* = 8) of respondents who reported the quality of hearing aids as “rather not sufficient” for making music. Consequently, many refrained from using them, with 33% (*N* = 5) reporting they never used hearing aids for music listening.

### Health-Related Quality of Life

**Figure 6.**
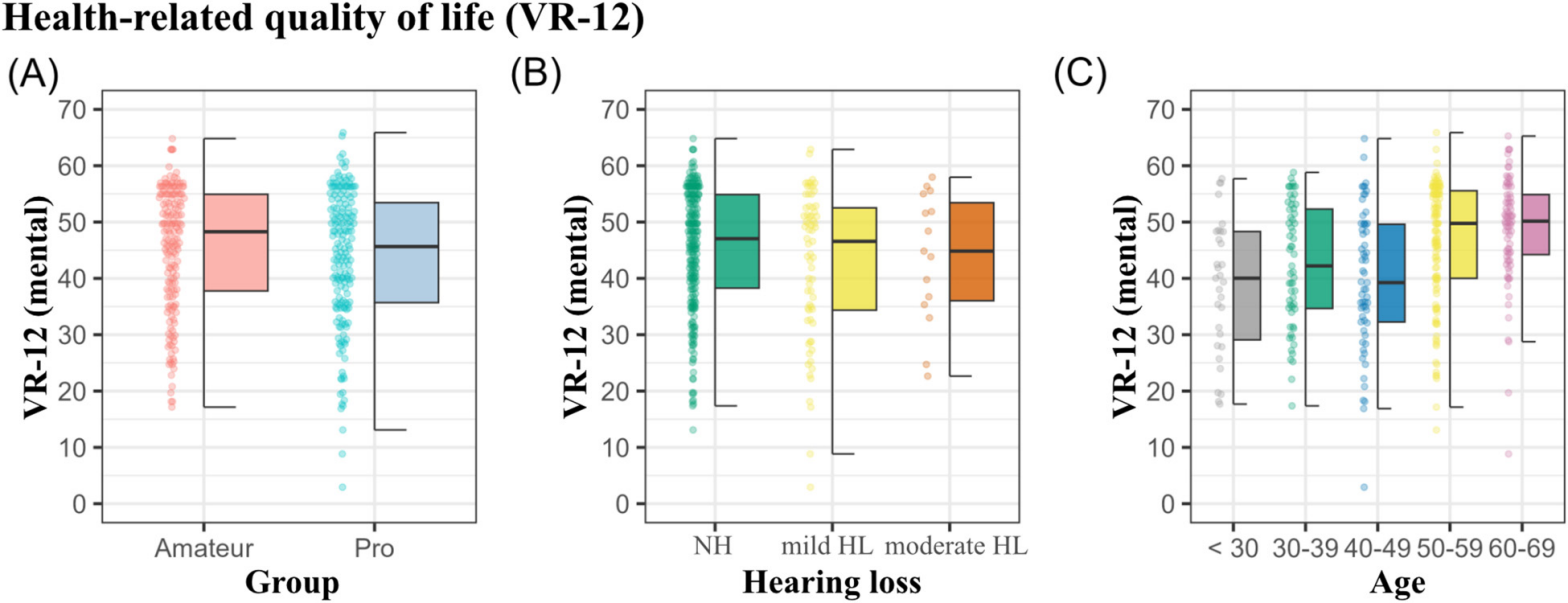
Health-related quality of life Veterans RAND 12-Item Health Survey (VR-12) as a function of group (A), HL (B), and age (C).

In the evaluation of veterans health-related quality of life scale (VR-12), no significant differences were observed between amateur and professional musicians with respect to the mental health component score, VR12-MCS; *W*(353) =  16,465, *p* = .35, see Figure 6, or the physical health component score, VR12-PCS; *W*(353) =  16,435, *p* = .37. Furthermore, no differences could be observed between different degrees of HL and the VR-12, *H*(4,364) = .58, *p* = .75. However, the mental health component score correlated with age, *r*(364) = .29, *p* < .001, with musicians below 30 years reporting the worst mean scores (*M* = 38.6, SD = 12), and the oldest age group of 60 + years the least inclination of mental quality of life issues (*M* = 48.9, SD = 9.5).

### Interscale Correlation Analysis

In the interscale correlation analysis, significant correlations among variables related to age, MRHPs, hearing in daily life, HHA, and health related quality of life indicators were found (for detailed correlation matrices, see Table 2). Specifically, a positive relationship was found between the UNE and HHA, *r*(330) = .24, *p* < .001, indicating that individuals with higher noise exposure report greater awareness of hearing health related issues. Furthermore, a relationship was observed between HHA and the HDDA subscale for social interactions, *r*(350) = −.32, *p* < .001, and with MRHP, *r*(345) = .20, *p* < .001. Besides that, MRHP was moderately correlated with the HDDA subscale for social interactions, *r*(365) = −.39, *p* < .001. These findings imply that difficulties in hearing, both in social and musical contexts, often co-occur and are associated with heightened awareness of hearing health. Intriguingly, however, the UNE did not significantly correlate with either the HDDA subscale for social interactions, *r*(355) = 0, *p* = .75 or MRHP, *r*(342) = −.08, *p* = .152. Lastly, there was a notable negative correlation between MRHP and the mental health component of quality of life, *r* = −.23, *p* < .001.

**Table 2.** The Correlation Matrix of Selected Tests from the Test Battery.

Variable	Age	UNE	LUNE	MRHP	HDDA (social)	HAD	HHA	VR12 (mental)
Age	—	−.08	.24***	−.01	−.22***	.05	−.02	−.3***
UNE	364	—	.91***	−.08	.02	.10	.24***	.01
LUNE	362	362	—	−.08	−.06	.14*	.27***	.12*
MRHP	374	344	342	—	−.39***	−.17**	.20**	−.24***
HDDA (social)	391	357	355	367	—	−.14*	−.32***	.20***
HAD	335	312	310	326	330	—	.31***	−.13*
HHA	359	330	328	347	352	317	—	−.13*
VR12 (mental)	353	325	323	340	346	314	332	—

*Note.* In the upper right half of the correlation matrix, the results are presented as Spearman's correlation coefficients (rho). The lower half of the matrix displays the respective sample sizes (*N*) used in each analysis. Significance levels are indicated as follows: *p < .05, **p < .01, and ***p < .001. Significant values are highlighted in bold.

## Discussion

A focal point of this investigation was to determine whether professional musicians would (a) report a greater incidence of hearing problems and (b) exhibit a heightened awareness toward hearing health compared to their amateur counterparts. To allow for a nuanced comparative analysis between these musicians, a carefully curated and balanced subsample of 200 professional and 200 amateur musicians, matched based on age, gender, and instrument family parameters was established.

### Noise Exposure and Musicians Hearing Problems

The comparative analysis highlighted notable discrepancies in weekly music exposure, with professionals averaging 30.9 hours and amateurs 7.4 hours of active playing. These figures align with prior findings, stating average practice times of 24–30 hours per week for professional musicians (e.g., Laitinen et al., 2003, Schmidt et al., 2011). The study considered estimates of sound level exposure across various playing settings, from solitary practice to full orchestral rehearsals, alongside the attenuation of music noise exposure via hearing protection usage, thereby establishing a measure for lifetime music noise exposure. The results revealed that professional musicians experience almost three times the yearly music sound exposure and nearly four times the lifetime exposure compared to their amateur counterparts. This significant disparity persists despite a higher occasional usage of hearing protection among professionals (53.5%) compared to amateurs (12%). Examining lifetime exposure, 50% of professional musicians, as opposed to ∼10% of amateurs, reached an exposure level equivalent to experiencing 90 dB-A for 8 hours daily, 5 days a week, over 52 weeks annually, for a decade. Such levels of exposure, accumulated solely through music playing, are alarming, as they mirror conditions known to cause permanent HL, as documented in various studies and guidelines (e.g., Imam & Hannan, 2017; Liedtke, 2010; see also “Königsteiner” and “NIOSH” recommendations; Chan, 1998; Deutsche Gesetzliche Unfallversicherung, 2013, 2020).

As a consequence of these high levels of music sound exposure, it was hypothesized that professional musicians would experience more severe hearing problems—both music-related and in daily life contexts—compared to amateurs. Indeed, two thirds of the musicians reported some kind of hearing problems. But despite the reported differences in musical engagement and its influence on music noise exposure levels, almost no group differences in reported hearing problems could be observed. Specifically, professional and amateur musicians reported comparable rates of self-reported HL (22% amateurs vs. 23% of professional), experiences with tinnitus (23.3% amateurs vs. 24.7% of professional), and oversensitivity to loud sound levels (44.8% amateurs vs. 53.3% of professional). Also scores from the MRHP scale and the subscore of the HDDA subscale for basic sounds did not differ significantly between professionals and amateurs. Furthermore, we could neither find an association of noise exposure with HDDA or MRHP, nor could we predict differences between the degree of HL with the music UNE. There was only one domain in which professional musicians reported significantly more severe hearing problems compared to amateurs: in the HDDA subscore for social interactions—with a rather small effect size.

The reported prevalence of hearing disorders among musicians, as found in this study, aligns with prior surveys (e.g., Harper, 2002; Hasson et al., 2009), yet the lack of a significant difference between professional and amateur musicians in terms of hearing impairments, despite differing levels of music noise exposure, is notable. This observation resonates with challenges faced by other researchers, such as Prendergast et al. (2017) and Russo et al. (2013), who also reported difficulties in establishing a straightforward link between levels of noise exposure and hearing impairments among musicians (see also Maghiar et al., 2023). Several reasons may account for these findings.

One possible explanation is that perceptual differences in hearing impairments that result from prolonged noise exposure may manifest gradually and too subtle for detection through self-report methods. Furthermore, musicianship has been linked to enhanced auditory abilities such as improved rhythm perception, pitch discrimination, and timbre identification (e.g., Grahn & Rowe, 2009; Micheyl et al., 2006; Nave-Blodgett et al., 2021; Tużnik et al., 2018; Zendel & Alain, 2012). There is even some evidence suggesting musical training may also benefit speech perception (Coffey et al., 2017), which is, however, still under debate (e.g., Madsen et al., 2019; McKay, 2021). Accordingly, musical training might be conceived to compensate for the negative effects of hearing impairments. Considering that two-thirds of the participants in this study are under 60 years of age, the subtle nature of noise-induced hearing changes, coupled with potential compensatory mechanisms developed through auditory training, could make early detection challenging. Thus, broad survey methods may not effectively capture these nuanced individual variations and contexts. Future studies should consider integrating hearing assessment methods, such as high-frequency extended pure-tone audiometry (e.g., Couth et al. 2020, 2024), to validate self-reported data and ensure more robust findings.

Another potential explanation of these findings could relate to the operationalization of the unit of UNE score. This metric, designed to provide a coarse estimate of prolonged noise exposure, is based on several assumptions that may affect its precision in identifying correlations with hearing problems. A key factor is the specific instrument and the repertoire played by musicians, as these can significantly impact sound exposure. For example, Schmidt et al. (2011) noted a substantial difference of 9.6 dB between repertoires, underscoring the repertoire as a significant factor in musicians’ exposure (also see Laitinen et al., 2003; Rodrigues et al., 2014). Moreover, the model adopts a conservative approach, considering a constant noise exposure during individual practice from the age of 10 and orchestral/rehearsal noise exposure from age 20. This cross-sectional method does not account for variations in playing times throughout a musician’s career and life, instead it presumes a constant exposure over time. Furthermore, the NESI examination instrument (Guest et al., 2018) used to calculate the musicians’ sound level exposure was originally designed to capture both occupational and recreational activities. However, in this study, it was only applied to sound exposure from instrumental music encountered during active music-making activities. Recreational music listening and other everyday sources of noise exposure were not considered. Couth et al. (2020) utilized the same instrument but included recreational activities to calculate the total lifetime noise exposure between musicians and nonmusicians (not specifically excluding amateur musicians), finding similar levels of exposure for both groups when recreational activities were included, as the majority of noise exposure could be attributed to recreational activities. Accordingly, in our sample, the musicians’ total lifetime noise exposure may be more similar when recreational activities are taken into account, which could explain the lack of differences in reported hearing problems between professional and amateur musicians. It is conceivable that employing more detailed estimates, which account for different career phases and other noise exposure contexts, might uncover effects that the current methodology does not detect. Still, recent work by Couth et al. (2024) also shows minimal effects on hearing from noise exposure utilizing a longitudinal design, suggesting that the effects observed in this study may remain stable over time.

Certainly, establishing a direct link between musical sound exposure and hearing impairments proves challenging (see also Couth et al., 2020; Elmazoska et al., 2024; Kähäri et al., 2001a; 2001b), which suggests that the risk of noise-induced HL in musicians might have been overestimated in previous research. Additionally, there is evidence that occupational noise, such as that encountered on construction sites, may be more detrimental to hearing than recreational sound, including music, possibly due to the dynamic nature of musical sounds (Neitzel & Fligor, 2019). Nonetheless, it is crucial to recognize that many musicians, both amateurs and professionals, are exposed to exceptionally high noise levels during their practice and performances. This exposure is particularly pronounced during individual practice sessions, where long uninterrupted playing and infrequent use of hearing protection are common. Thus, in sum, these findings underscore the importance of preventive measures to preserve musicians’ hearing health.

### Preventive Measures and Hearing Health Awareness

While hearing protection devices offer great potential for improving musicians’ hearing health, their utilization remains alarmingly low. The analysis showed greater (occasional) usage of hearing protection among professional musicians (53.5%) compared to amateurs (12%). This is evident despite 73% of professionals reporting that hearing protection impedes their performance; yet, musicians continue its usage, demonstrating a complex balance between auditory health and musical quality. The majority of musicians who use hearing protection opt for customized solutions (professionals: 84%; amateurs: 65%), indicating an understanding of the importance of personalized fit and comfort. However, a concerningly low 1% of amateur musicians’ playing hours and only 9% of professional musicians’ playing hours are fully protected. In contrast, 80% of amateurs’ and 36% of professionals’ playing hours remain completely unprotected. Given the potential benefits of hearing protection (e.g., Kwak & Han, 2021), these numbers underscore the need for action to promote greater hearing protection adoption within the music community. Intriguingly, musicians demonstrate a pattern of increasing HHA as their hearing problems worsen (as also noted by Laitinen, 2005). This negative relationship exists in both music-related settings and social interactions and is mirrored in hearing protection usage: only 3% of those with NH use full protection, increasing to 5% for those with mild HL and a notable 22% for those with at least moderate HL. These findings support the “reactive action” phenomenon, where proactive measures are often taken only after issues arise.

The low utilization rate of hearing protection resonates with findings from nearly two decades ago, as reported by Laitinen (2005), where 6% of Finnish musicians were noted to always use hearing protection devices. The stagnation in the adoption of protective measures, even considering variations among different countries, underscores the necessity for effective strategies and interventions that are specifically tailored to the unique needs of the music community. Several factors influence the notably low uptake of hearing protection across the musician community. On the one hand, legislative measures play a crucial role; an overwhelming majority of professionals (97%) regularly undergo hearing checks, typically every three years, as contrasted with 70% of amateurs, who tend to seek audiologist consultations only once every seven years on average. Accordingly, such regulatory mandates, as outlined in the German Noise and Vibrations—Occupational Safety and Health Ordinance (2007), are likely central in promoting higher rates of hearing protection use among professional musicians. Performance-related anxieties, on the other hand, contribute to the reluctance in adopting hearing protection; 80% of professionals and 32% of amateurs who refrained from using hearing protection said they refrained from using hearing protection because they are concerned their performance might suffer. However, the primary factor is the lack of awareness about hearing health issues. On average, amateur musicians exhibited considerably lower average awareness scores compared to professional musicians. This discrepancy is most evident among younger musicians who, despite facing higher music noise exposure, are least inclined to use hearing protection. This finding contrasts with the report by Couth et al. (2021), which indicated that 77% of young musicians employ hearing protection at least once a week. Intriguingly, young musicians (<30 years) in this study had the highest awareness scores, prompting a re-evaluation of the direct association between awareness and behavioral change as proposed by Ajzen (1991) and underscores the complexity of translating awareness into action.

In conclusion, while establishing a clear link between heightened awareness of hearing health and the consistent use of hearing protection proves challenging, expanding regulatory measures to encompass amateur orchestras could offer substantial benefits. Furthermore, addressing performance-related anxieties and providing information on adaptation processes to mitigate performance impacts could be pivotal components of awareness programs (see also Couth et al., 2021 for model strategies).

### Hearing aid Usage

A mere fraction of musicians across both professional and amateur settings (N = 15) reported continuing usage of hearing aids, highlighting a noteworthy yet anticipated low utilization rate. Furthermore, 54% of these users expressed that their hearing aids are not sufficient for making music, and only 80% reported that hearing aids improve their hearing at all. This finding aligns with existing literature indicating that current hearing aid technologies may not sufficiently cater to the specialized auditory needs of musicians: Previous survey studies have demonstrated that although hearing aids were perceived as useful in listening to both live and recorded music, their efficacy was deemed less satisfactory for live musical settings—a context of paramount importance for musicians. Common issues reported include distortion, acoustic feedback, imbalanced gain, and compromised tone quality (Madsen & Moore, 2014). However, it is essential to acknowledge more recent studies presenting a more favorable view. These suggest that hearing aid usage can indeed improve music enjoyment and appreciation for both speech and music (Chern et al., 2023).

One major concern in this research centers on the potential underrepresentation of individuals with severe hearing impairments who have found hearing aids to be ineffective for musical tasks. Such individuals may have withdrawn from musical activities altogether, thereby becoming statistically invisible within the current study sample. Indeed, more than half of the respondents who reported having tried hearing aids in the past (16 out of 31) refrained from using them. This phenomenon may correspond to an instance of “survivorship bias,” an analytical error characterized by an undue focus on subjects that “survive” a given process while overlooking those who do not. Those subjects who report finding hearing aids effective are likely those who have persisted in musical activities despite hearing challenges. This could inadvertently exclude the experiences of those who have ceased musical involvement due to the inadequacy of hearing aids for music-related tasks. The recognition of survivorship bias and its potential impact on the study’s findings has significant implications for this research's validity (that is, the reported utility rating are likely an overestimation of the usefulness of hearing aids), but also has implications for future investigative directions: Subsequent studies could adopt sampling techniques specifically designed to capture the experiences of individuals who have withdrawn from musical activities due to the limitations of hearing aids and hearing impairments. By doing so, a more comprehensive and accurate understanding of the true effectiveness and limitations of hearing aids may be attained, especially in the domain of music perception and performance.

Our findings further underscore the pivotal role of attitudes toward hearing aids as a moderator in the adoption and sustained utilization of hearing aids among musicians. In our study, professional musicians manifested a markedly higher degree of disapproval toward HAD compared to their amateur counterparts. Moreover, one out of four musicians indicated hesitancy in openly discussing their HL in public. This differential disapproval may be anchored in both the stigmatization of hearing aids and their perceived functional limitations within professional musical settings. The constructed scale for HAD encapsulates multiple facets that resonate with this notion, including irritation caused by colleagues wearing hearing aids, reluctance toward nondiscrete devices, apprehensions about diminished peer respect, adverse audience perceptions, and the presumed incongruence between professional musicianship and hearing aid use. These elements collectively converge to form a pervasive stigma surrounding hearing aids, particularly within the realm of professional musicianship. The implications of this aversion are twofold. First, it elucidates an unexplored barrier to hearing aid adoption among a specialized population. Second, it offers a fertile ground for targeted interventions designed to mitigate these attitudes, thereby potentially augmenting hearing aid uptake and sustained usage among professional musicians. Additional empirical inquiries are imperative for a conclusive understanding of the dimensions and implications of hearing aid aversion in this particular cohort.

Importantly, the distribution of HAD among musicians presents a bimodal pattern. While a considerable majority exhibits minimal disapproval, a notable subgroup demonstrates substantial disapproval of hearing aids. There is some indication that older individuals tend to express higher disapproval toward hearing aids, yet a straightforward relationship could not be observed. Certainly, age-dependent inclination toward hearing aid aversion merits further empirical scrutiny, as it could have broader implications for targeted interventions aimed at enhancing hearing aid adoption among ageing musicians.

### Musicians’ Overall Well-Being

No differences in terms of the health-related quality of life scale could be observed between professional and amateur musicians. The overall state of both physical and mental health in our sample of amateur and professional musicians can be characterized as healthy. This claim is substantiated when comparing published benchmark values from populations undergoing healthcare challenges. For instance, Buchholz et al. (2017) reported mean scores of 33 on the physical sum scale and 41 on the mental sum scale in a population of patients undergoing orthopedic rehabilitation. Similarly, a study by Hüppe et al. (2022) involving patients with chronic pain reflected comparable results with average scores of 28.1 and 37.6 on the physical and mental scales respectively. In comparison, musicians from this study’s sample demonstrates a substantially healthier profile, which aligns more closely with scores anticipated from a generally healthy population. However, there is considerable variability in the subscale for mental health among participants, with 113 musicians scoring below 40 (comparable to orthopedic rehabilitation; see Buchholz et al., 2017) and 48 scoring below 30 (comparable to chronic pain; see Hüppe et al., 2022). Notably, a significant proportion of those with lower scores are in the youngest age group, indicating that younger individuals tend to have poorer scores on mental health. This is consistent with previous research into musicians’ psychological well-being; for example, Kenny et al. (2014) found that the youngest (female) musicians often report the highest levels of performance-related anxiety. Despite this, an increase in hearing issues related to music and in daily-life was associated with a decline in mental health-related quality of life, suggesting a significant psychological toll from these impairments.

### Limitations

Several limitations must be acknowledged to contextualize the present findings. First, despite the efficacy of the matching procedure in equalizing pivotal variables such as age, gender, and instrument type, additional imbalances—such as the differences in repertoire played—could not be completely controlled for. Second, the process of matching inevitably led to the exclusion of certain participants, thus reducing the sample size. Nevertheless, the remaining sample (N = 400) was sufficiently robust to yield statistically meaningful insights. Importantly, the matching strategy served to better isolate the effects of the variables of interest by minimizing potential bias due to confounding variables, thereby justifying the concomitant reduction in statistical power.

A potential self-selection bias poses a concern for the generalizability of the results. The voluntary nature of participation suggests that the sample might disproportionately represent musicians with a particular interest in hearing health, possibly due to personal experiences with hearing impairments. Consequently, the findings may exhibit a bias toward greater HHA, or a higher incidence of hearing issues compared to the general population of musicians, thereby limiting the generalizability of the results. However, it should be noted that many of the findings align with prior research on hearing health among musicians, not just in Germany but also in countries such as Australia and Finland, yielding corroborative evidence to the present study.

Additionally, the study’s reliance on self-reported data introduces another layer of complexity. For example, existing literature suggests that participants in survey research often overstate socially desirable behaviors (e.g., Tourangeau & Yan, 2007), thereby introducing measurement error. This remains uncontrolled and might induce bias, particularly among musicians who are acutely aware of their auditory capabilities and may report subtle issues irrespective of actual noise exposure. Nevertheless, this propensity for “over-reporting” is more pronounced in interviewer-administered surveys than in self-administered questionnaires, especially when the latter are conducted anonymously and voluntarily. Hence, while the present study has attempted to mitigate this bias through its design, the possibility of misrepresentation of certain attitudes still warrants cautious interpretation of the findings.

## Conclusion

This study sheds light on the intricate relationship between musicians’ unique hearing health challenges, attitudes, and preventive behaviors in German-speaking orchestras. Contrary to initial assumptions, professional musicians did not universally experience higher levels of hearing-related issues, despite encountering significantly greater levels of music-related noise exposure. However, these musicians were more proactive in adopting preventative measures such as hearing protection devices. This proactivity did not extend to hearing aid adoption, as professionals displayed noticeable aversion to these devices—an aversion that appears to be partly age-dependent. In this regard, amateur musicians exhibited considerably lower aversion toward hearing aids while showing lower general awareness of hearing health. By highlighting the differences in exposure and preventive actions between professional and amateur musicians, the present study underscores the need for tailored hearing health strategies by specifically suggesting a more proactive and informed approach to the preservation of hearing health within the musical community.

## Supplemental Material

sj-docx-1-tia-10.1177_23312165241293762 - Supplemental material for A Survey on Hearing Health of Musicians in Professional and Amateur Orchestras Supplemental material, sj-docx-1-tia-10.1177_23312165241293762 for A Survey on Hearing Health of Musicians in Professional and Amateur Orchestras by Robin Hake, Gunter Kreutz, Ulrike Frischen, Merle Schlender, Esther Rois-Merz, Markus Meis, Kirsten C. Wagener and Kai Siedenburg in Trends in Hearing

## References

[bibr1-23312165241293762] Ajzen I. (1991). The theory of planned behavior. Organizational Behavior and Human Decision Processes, 50(2), 179–211. 10.1016/0749-5978(91)90020-T

[bibr2-23312165241293762] Bigelow R. T. Reed N. Brewster K. K. Huang A. Rebok G. W. Rutherford B. R. Lin F. R. (2020). Association of hearing loss with psychological distress and utilization of mental health services among adults in the United States. JAMA Network Open, 3(7), e2010986. 10.1001/jamanetworkopen.2020.10986 PMC7372323 32687587

[bibr3-23312165241293762] Buchholz I. Kohlmann T. Buchholz M. (2017). Vergleichende Untersuchung der psychometrischen Eigenschaften des SF-36/SF-12 vs. VR-36/VR-12. *Abschlussbericht*.

[bibr4-23312165241293762] Chan H. S. (1998). Occupational noise exposure: Criteria for a recommended standard (DHHS (NIOSH) Publication No. 98-126). *National Institute for Occupational Safety and Health*. https://stacks.cdc.gov/view/cdc/6376

[bibr5-23312165241293762] Chern A. Denham M. W. Leiderman A. S. Sharma R. K. Su I. W. Ucci A. J. Jones J. M. Mancuso D. Cellum I. P. Galatioto J. A. Lalwani A. K. (2023). The association of hearing loss with active music enjoyment in hearing aid users. Otolaryngology–Head and Neck Surgery, 169(6), 1590–1596. 10.1002/ohn.473 37555237

[bibr6-23312165241293762] Coffey E. B. J. Mogilever N. B. Zatorre R. J. (2017). Speech-in-noise perception in musicians: A review. Hearing Research, 352, 49–69. 10.1016/j.heares.2017.02.006 28213134

[bibr7-23312165241293762] Couth S. Loughran M. T. Plack C. J. Moore D. R. Munro K. J. Ginsborg J. Dawes P. Armitage C. J. (2021). Identifying barriers and facilitators of hearing protection use in early-career musicians: A basis for designing interventions to promote uptake and sustained use. International Journal of Audiology, 61(6), 463–472. 10.1080/14992027.2021.1951852 34406107

[bibr8-23312165241293762] Couth S. Prendergast G. Guest H. Munro K. J. Moore D. R. Plack C. J. Ginsborg J. Dawes P. (2020). Investigating the effects of noise exposure on self-report, behavioral and electrophysiological indices of hearing damage in musicians with normal audiometric thresholds. Hearing Research, 395, 108021. 10.1016/j.heares.2020.108021 32631495

[bibr9-23312165241293762] Couth S. Prendergast G. Guest H. Munro K. J. Moore D. R. Plack C. J. Ginsborg J. Dawes P. (2024). A longitudinal study investigating the effects of noise exposure on behavioural, electrophysiological and self-report measures of hearing in musicians with normal audiometric thresholds. Hearing Research, 451, 109077. 10.1016/j.heares.2024.109077 39084132

[bibr10-23312165241293762] Deutsche Gesetzliche Unfallversicherung. (2013). *Präventionsleitlinie “Gehörschutz für Musiker”*. https://www.dguv.de/medien/fb-psa/de/sachgebiet/sg_gehoerschutz/praevleit_musik.pdf

[bibr11-23312165241293762] Deutsche Gesetzliche Unfallversicherung. (2020). *Empfehlung für die Begutachtung der Lärmschwerhörigkeit* (*BK-Nr. 2301*) – *Königsteiner Empfehlung – Update* 2020. Dr. Ulrike Wolf & Stefanie Palfner (Redaktion). https://www.dguv.de/publikationen

[bibr12-23312165241293762] Deutsches Musikinformationszentrum. (2021). *Amateurmusizieren in Deutschland: Ergebnisse einer Repräsentativbefragung in der Bevölkerung ab 6 Jahre*. Institut für Demoskopie Allensbach, Deutscher Musikrat, Bonn. https://bundesmusikverband.de/zahlen/

[bibr13-23312165241293762] Dingle G. A. Sharman L. S. Bauer Z. Beckman E. Broughton M. Bunzli E. Davidson R. Draper G. Fairley S. Farrell C. Flynn L. M. Gomersall S. Hong M. Larwood J. Lee C. Lee J. Nitschinsk L. Peluso N. Reedman S. E. Wright O. R. L. (2021). How do music activities affect health and well-being? A scoping review of studies examining psychosocial mechanisms. Frontiers in Psychology, 12, 713818. 10.3389/fpsyg.2021.713818 34566791 PMC8455907

[bibr14-23312165241293762] Di Stadio A. Dipietro L. Ricci G. Della Volpe A. Minni A. Greco A. de Vincentiis M. Ralli M. (2018). Hearing loss, tinnitus, hyperacusis, and diplacusis in professional musicians: A systematic review. International Journal of Environmental Research and Public Health, 15(10), 2120. 10.3390/ijerph15102120 30261653 PMC6209930

[bibr15-23312165241293762] Elmazoska I. Mäki-Torkko E. Granberg S. Widén S. (2024). Associations between recreational noise exposure and hearing function in adolescents and young adults: A systematic review. Journal of Speech, Language, and Hearing Research : JSLHR, 67(2), 688–710. 10.1044/2023_JSLHR-23-00397 38324255

[bibr16-23312165241293762] Gembris H. Heye A. Seifert A. (2018). Health problems of orchestral musicians from a life-span perspective: Results of a large-scale study. Music and Science, 1, 1–20. 10.1177/2059204317739801

[bibr17-23312165241293762] Gong R. Hu X. Gong C. Long M. Han R. Zhou L. Wang F. Zheng X. (2018). Hearing loss prevalence and risk factors among older adults in China. International Journal of Audiology, 57(5), 354–359. 10.1080/14992027.2017.1423404 29400111

[bibr18-23312165241293762] Grahn J. A. Rowe J. B. (2009). Feeling the beat: Premotor and striatal interactions in musicians and nonmusicians during beat perception. The Journal of Neuroscience : The Official Journal of the Society for Neuroscience, 29(23), 7540–7548. 10.1523/JNEUROSCI.2018-08.2009 19515922 PMC2702750

[bibr19-23312165241293762] Greasley A. E. (2022). Diverse music listening experiences: Insights from the hearing aids for music project. In Drever J. Hugill A. (Eds.), Aural diversity (pp. 134–142). Routledge.

[bibr20-23312165241293762] Greasley A. E. Fulford R. J. Pickard M. Hamilton N. (2020). Help musicians UK hearing survey: Musicians’ hearing and hearing protection. Psychology of Music, 48(4), 529–546. 10.1177/0305735618812238

[bibr21-23312165241293762] Guest H. Dewey R. S. Plack C. J. Couth S. Prendergast G. Bakay W. Hall D. A. (2018). The Noise Exposure Structured Interview (NESI): An instrument for the comprehensive estimation of lifetime noise exposure. Trends in Hearing, 22, 10.1177/2331216518803213 PMC6176535 30295145

[bibr22-23312165241293762] Hake R. Kreutz G. Frischen U. Schlender M. Rois-Merz E. Meis M. Wagener K. C. Siedenburg K. (2024). Umfrage zur Hörgesundheit von Ensembleleitung, Berufs- und Amateurmusikerinnen in deutschsprachigen Orchestern und Chören [Data]. GESIS, Cologne. Data File Version 1.0.0. 10.7802/2695 PMC11653104 39648735

[bibr23-23312165241293762] Halevi-Katz D. Yaakobi E. Putter-Katz H. (2015). Exposure to music and noise-induced hearing loss (NIHL) among professional pop/rock/jazz musicians. Noise and Health, 17(76), 158–164. 10.4103/1463-1741.155848 25913555 PMC4918652

[bibr24-23312165241293762] Harper B. S. (2002). Workplace and health: A survey of classical orchestral musicians in the United Kingdom and Germany. Medical Problems of Performing Artists, 17, 83–92. 10.21091/mppa.2002.2012

[bibr25-23312165241293762] Hasson D. Theorell T. Liljeholm-Johansson Y. Canlon B. (2009). Psychosocial and physiological correlates of self- reported hearing problems in male and female musicians in symphony orchestras. International Journal of Psychophysiology, 74, 93–100. 10.1016/j.ijpsycho.2009.07.009 19666059

[bibr26-23312165241293762] Hébert S. Lupien S. J. (2009). Salivary cortisol levels, subjective stress, and tinnitus intensity in tinnitus sufferers during noise exposure in the laboratory. International Journal of Hygiene and Environmental Health, 212(1), 37–44. 10.1016/j.ijheh.2007.11.005 18243788

[bibr27-23312165241293762] Hidalgo J. L. T. Gras C. B. Lapeira J. M. T. Martínez I. P. Verdejo MÁL Rabadán F. E. Puime ÁO (2008). The hearing-dependent daily activities scale to evaluate impact of hearing loss in older people. Annals of Family Medicine, 6(5), 441–447. 10.1370/afm.890 18779549 PMC2532761

[bibr28-23312165241293762] Ho D. E. Imai K. King G. Stuart E. A. (2007). Matching as nonparametric preprocessing for reducing model dependence in parametric causal inference. Political Analysis, 15(3), 199–236. 10.1093/pan/mpl013

[bibr29-23312165241293762] Humes L. E. (2019). The World Health Organization's hearing-impairment grading system: An evaluation for unaided communication in age-related hearing loss. International Journal of Audiology, 58(1), 12–20. 10.1080/14992027.2018.1518598 30318941 PMC6351193

[bibr30-23312165241293762] Hüppe M. Schneider K. Casser H. R. Knille A. Kohlmann T. Lindena G. Nagel B. Nelles J. Pfingsten M. Petzke F. (2022). Characteristic values and test statistical goodness of the Veterans RAND 12-Item Health Survey (VR-12) in patients with chronic pain: An evaluation based on the KEDOQ pain dataset. Schmerz, 36(2), 109–120. 10.1007/s00482-021-00570-5 34279750 PMC8956556

[bibr31-23312165241293762] Imam L. Hannan S. A. (2017). Noise-induced hearing loss: A modern epidemic? British Journal of Hospital Medicine, 78(5), 286–290. 10.12968/hmed.2017.78.5.286 28489444

[bibr32-23312165241293762] Jacob R. Stelzig Y. Buschermöhle M. Berg D. Meis M. (2017). Questionnaires as screening tools to identify persons with hearing deficiencies. Zeitschrift fur Audiologie, 56(3), 103–108.

[bibr33-23312165241293762] Kähäri K. R. Axelsson A. Hellström P.-A. Zachau G. (2001a). Hearing assessment of classical orchestral musicians. Scandinavian Audiology, 30, 13–23. 11330914 10.1080/010503901750069536

[bibr34-23312165241293762] Kähäri K. R. Axelsson A. Hellström P.-A. Zachau G. (2001b). Hearing development in classical orchestral musicians: A follow-up study. Scandinavian Audiology, 30, 141–149. 10.1080/010503901316914511 11683452

[bibr35-23312165241293762] Kenny D. Driscoll T. Ackermann B. (2014). Psychological well-being in professional orchestral musicians in Australia: A descriptive population study. Psychology of Music, 42(2), 210–232. 10.1177/0305735612463950

[bibr36-23312165241293762] Keppler H. Dhooge I. Vinck B. (2015a). Hearing in young adults. Part I: The effects of attitudes and beliefs toward noise, hearing loss, and hearing protector devices. Noise & Health, 17(78), 237–244. 10.4103/1463-1741.165024 26356365 PMC4900495

[bibr37-23312165241293762] Keppler H. Dhooge I. Vinck B. (2015b). Hearing in young adults. Part II: The effects of recreational noise exposure. Noise & Health, 17(78), 245–252. 10.4103/1463-1741.165026 26356366 PMC4900507

[bibr38-23312165241293762] Keppler H. Ingeborg D. Sofie D. Bart V. (2015C). The effects of a hearing education program on recreational noise exposure, attitudes and beliefs toward noise, hearing loss, and hearing protector devices in young adults. Noise & Health, 17(78), 253–262. 10.4103/1463-1741.165028 26356367 PMC4900500

[bibr39-23312165241293762] Kerby D. S. (2014). The simple difference formula: An approach to teaching nonparametric correlation. Comprehensive Psychology, 3, 11.IT.3.1. 10.2466/11.IT.3.1

[bibr41-23312165241293762] Kwak C. Han W. (2021). The effectiveness of hearing protection devices: A systematic review and meta-analysis. International Journal of Environmental Research and Public Health, 18(21), 11693. 10.3390/ijerph182111693 34770206 PMC8583416

[bibr42-23312165241293762] Laitinen H. (2005). Factors affecting the use of hearing protectors among classical music players. Noise & Health, 7, 21–29. 10.4103/1463-1741.31643 16053602

[bibr43-23312165241293762] Laitinen H. M. Toppila E. M. Olkinuora P. S. Kuisma K. (2003). Sound exposure among the Finnish National Opera personnel. Applied Occupational and Environmental Hygiene, 18(3), 177–182. 10.1080/10473220301356 12573963

[bibr44-23312165241293762] Leiner D. J. (2019). *SoSci survey* (Version 3.1.06) [Computer software]. https://www.soscisurvey.de

[bibr45-23312165241293762] Liedtke M. (2010). Akute Gehörschäden durch extrem hohe Schalldruckpegel. HNO, 58(2), 106–109. 10.1007/s00106-009-2059-0 20127065

[bibr46-23312165241293762] Madsen S. M. K. Marschall M. Dau T. Oxenham A. J. (2019). Speech perception is similar for musicians and non-musicians across a wide range of conditions. Scientific Reports, 9(1), 1–10. 10.1038/s41598-019-46728-1 31320656 PMC6639310

[bibr47-23312165241293762] Madsen S. M. K. Moore B. C. J. (2014). Music and hearing aids. Trends in Hearing, 18, 10.1177/2331216514558271 PMC4271761 25361601

[bibr48-23312165241293762] Maghiar M. J. Lawrence B. J. Mulders W. H. A. M. Moyle T. C. Livings I. Jayakody D. M. P. (2023). Hearing loss and mental health issues in amateur and professional musicians. Psychology of Music, 51(6), 1584–1597. 10.1177/03057356231155970

[bibr49-23312165241293762] Martini A. E. (1996). European Working Group on genetics of hearing impairment. European Commission Directorate, Biomedical and Health Research Programme (HEAR). Infoletter, 2, 8.

[bibr50-23312165241293762] McKay C. M. (2021). No evidence that music training benefits speech perception in hearing-impaired listeners: A systematic review. Trends in Hearing, 25, 233121652098567. 10.1177/2331216520985678 PMC7934028 33634750

[bibr51-23312165241293762] Micheyl C. Delhommeau K. Perrot X. Oxenham A. J. (2006). Influence of musical and psychoacoustical training on pitch discrimination. Hearing Research, 219(1-2), 36–47. 10.1016/j.heares.2006.05.004 16839723

[bibr52-23312165241293762] Müllensiefen D. Gingras B. Musil J. Stewart L. (2014). The musicality of non-musicians: An index for assessing musical sophistication in the general population. PLoS ONE, 9(2), e89642. 10.1371/journal.pone.0089642 PMC3935919 24586929

[bibr53-23312165241293762] Musgrave G. (2022). Music and wellbeing vs. musicians’ wellbeing: Examining the paradox of music-making positively impacting wellbeing, but musicians suffering from poor mental health. Cultural Trends, 32(3), 280–295. 10.1080/09548963.2022.2058354

[bibr54-23312165241293762] Nave-Blodgett J. E. Snyder J. S. Hannon E. E. (2021). Hierarchical beat perception develops throughout childhood and adolescence and is enhanced in those with musical training. Journal of Experimental Psychology. General, 150(2), 314–339. 10.1037/xge0000903 32852978

[bibr55-23312165241293762] Neitzel R. L. Fligor B. J. (2019). Risk of noise-induced hearing loss due to recreational sound: Review and recommendations. The Journal of the Acoustical Society of America, 146(5), 3911–3921. 10.1121/1.5132287 31795675

[bibr56-23312165241293762] Noise and vibrations – Occupational Safety and Health Ordinance (2007) *Federal Law Gazette I from 6 March 2007 (*p*. 261), last amended by Article 3 of the Ordinance of 19 July 2010 (Federal Law Gazette I p. 960)*. https://www.gesetze-im-internet.de/l_rmvibrationsarbschv/

[bibr57-23312165241293762] O'Brien I. Ackermann B. J. Driscoll T. (2014). Hearing and hearing conservation practices among Australia's professional orchestral musicians. Noise and Health, 16(70), 189–195. 10.4103/1463-1741.134920 24953885

[bibr58-23312165241293762] O’Brien I. Driscoll T. Ackermann B. (2013). Sound exposure of professional orchestral musicians during solitary practice. The Journal of the Acoustical Society of America, 134(4), 2748–2754. 10.1121/1.4820900 24116413

[bibr59-23312165241293762] Occupational Safety and Health Administration. (2002). *Hearing conservation [OSHA 3074 2002 (Revised)]*. U.S. Department of Labor. https://www.osha.gov/sites/default/files/publications/osha3074.pdf

[bibr60-23312165241293762] Olsen A. D. Gooding L. F. Shikoh F. Graf J. (2016). Hearing health in college instrumental musicians and prevention of hearing loss. Medical Problems of Performing Artists, 31(1), 29–36. 10.21091/mppa.2016.1006 26966962

[bibr61-23312165241293762] Phillips S. L. Mace S. (2008). Sound level measurements in music practice rooms. Music Performance Research, 2(1993), 36–47.

[bibr62-23312165241293762] Prendergast G. Millman R. E. Guest H. Munro K. J. Kluk K. Dewey R. S. Hall D. A. Heinz M. G. Plack C. J. (2017). Effects of noise exposure on young adults with normal audiograms II: Behavioral measures. Hearing Research, 356, 74–86. 10.1016/j.heares.2017.10.007 29126651 PMC5714059

[bibr63-23312165241293762] Richter B. (2011). Hals-Nasen-Ohren-Heilkunde. In Spahn C. Richter B. Altenmüller E. (Eds.), Musikermedizin. Diagnostik, therapie und pravention von musikerspezifischen erkrankungen (pp. 271–290). Schattauer.

[bibr64-23312165241293762] Rodrigues M. A. Freitas M. A. Neves M. P. Silva M. V. (2014). Evaluation of the noise exposure of symphonic orchestra musicians. Noise & Health, 16(68), 40–46. 10.4103/1463-1741.127854 24583679

[bibr65-23312165241293762] Rosseel Y. (2012). Lavaan: An R package for structural equation modeling. Journal of Statistical Software, 48(2), 1–36. 10.18637/jss.v048.i02

[bibr66-23312165241293762] RStudio Team (2020). RStudio: Integrated Development for R. RStudio, PBC. http://www.rstudio.com/

[bibr67-23312165241293762] Russo F. A. Behar A. Chasin M. Mosher S. (2013). Noise exposure and hearing loss in classical orchestra musicians. International Journal of Industrial Ergonomics, 43(6), 474–478. 10.1016/j.ergon.2012.11.001

[bibr69-23312165241293762] Scherer K. R. (2004). Which emotions can be induced by music? What are the underlying mechanisms? And how can we measure them? Journal of New Music Research, 33(3), 239–251. 10.1080/0929821042000317822

[bibr70-23312165241293762] Schink T. Kreutz G. Busch V. Pigeot I. Ahrens W. (2014). Incidence and relative risk of hearing disorders in professional musicians. Occupational and Environmental Medicine, 71(7), 472–476. 10.1136/oemed-2014-102172 24790053 PMC4078669

[bibr71-23312165241293762] Schmidt J. H. Pedersen E. R. Juhl P. M. Christensen-Dalsgaard J. Andersen T. D. Poulsen T. Bælum J. (2011). Sound exposure of symphony orchestra musicians. Annals of Occupational Hygiene, 55(8), 893–905. 10.1093/annhyg/mer055 21841154

[bibr72-23312165241293762] Tourangeau R. Yan T. (2007). Sensitive questions in surveys. Psychological Bulletin, 133(5), 859–883. 10.1037/0033-2909.133.5.859 17723033

[bibr73-23312165241293762] Tufts J. B. Skoe E. (2018). Examining the noisy life of the college musician: Weeklong noise dosimetry of music and non-music activities. International Journal of Audiology, 57(Suppl. 1), S20–S27. 10.1080/14992027.2017.1405289 29172785

[bibr74-23312165241293762] Tużnik P. Augustynowicz P. Francuz P. (2018). Electrophysiological correlates of timbre imagery and perception. International Journal of Psychophysiology: Official Journal of the International Organization of Psychophysiology, 129, 9–17. 10.1016/j.ijpsycho.2018.05.004 29758238

[bibr75-23312165241293762] Versorgungsanstalt der deutschen Kulturorchester. (2021). *Bericht über das Geschäftsjahr* 2021. https://www.orchesterversorgung.de

[bibr76-23312165241293762] Wesseldijk L. W. Mosing M. A. Ullén F. (2020). Why is an early start of training related to musical skills in adulthood? A genetically informative study. Psychological Science, 32(1), 3–13. 10.1177/0956797620959014 33308000 PMC7809336

[bibr77-23312165241293762] World Health Organization. (2001). *International classification of functioning, disability and health*. Geneva, Switzerland: WHO.

[bibr78-23312165241293762] Zander M. F. Spahn C. Richter B. (2008). Employment and acceptance of hearing protectors in classical symphony and opera orchestras. Noise & Health, 10, 14–26. 10.4103/1463-1741.39004 18270404

[bibr79-23312165241293762] Zendel B. R. Alain C. (2012). Musicians experience less age-related decline in central auditory processing. Psychology and Aging, 27(2), 410–417. 10.1037/a0024816 21910546

[bibr80-23312165241293762] Zentner M. Grandjean D. Scherer K. R. (2008). Emotions evoked by the sound of music: Characterization, classification, and measurement. Emotion, 8(4), 494–521. 10.1037/1528-3542.8.4.494 18729581

[bibr81-23312165241293762] Zhang J. D. Schubert E. (2019). A single item measure for identifying musician and nonmusician categories based on measures of musical sophistication. Music Perception, 36(5), 457–467. 10.1525/mp.2019.36.5.457

