# Supplementary material for: A Survey on Hearing Health of Musicians in Professional and Amateur Orchestras
Source: Trends Hear. 2024 Dec 9;28:23312165241293762. doi: 10.1177/23312165241293762 (PMC11653104; doi:10.1177/23312165241293762)
Supplement: sj-docx-1-tia-10.1177_23312165241293762 - Supplemental material for A Survey on Hearing Health of Musicians in Professional and Amateur Orchestras [file sj-docx-1-tia-10.1177_23312165241293762.docx]

**Supplementary Material**

**Tables**

**Table A1**

*Self-assessment of individual’ hearing impairment*

| **Question** | | | | | | |  |
| --- | --- | --- | --- | --- | --- | --- | --- |
| Do you feel that you have a hearing loss? | | | | | | |  |
| *(While this may not map onto your experience exactly, which of these best describes your hearing without hearing aids.)* | | | | | | |  |
|  |  |  |  |  |  |  |  |
| **Options** | | | | | | |  |
| □ No, I do not feel that I have a hearing loss. | | | | | | |  |
| □ Yes, I have the feeling of being mildly hearing impaired (hearing loss of 20-40 dB): When I am talking to a person in a quiet room, I can usually understand a conversation. In noisy situations (e.g. in a pub) and in group conversations, I sometimes have problems understanding speech. | | | | | | |  |
| □ Yes, I have the feeling of being moderately hearing impaired (hearing loss of 41-60 dB):   I have difficulty understanding speech and often have to ask others to repeat what they have said, both in face-to-face conversations and on the phone. I find it difficult to follow conversations in noisy situations. I have to turn up the TV and radio to hear them clearly. | | | | | | |  |
| □ Yes, I have the feeling of being severely hearing impaired (hearing loss of 61-80 dB):  I can barely understand speech without hearing aids and use lip-reading/sign language to assist communication. | | | | | | |  |
| □ Yes, I have the feeling of being profoundly hearing impaired (hearing loss bordering on deafness; 81-95 dB): Ordinary conversations are unintelligible. I cannot hear shouted words and have to rely on lip reading and/or sign language to communicate. | | | | | | |  |
| □ I don’t know | | | | | | |  |

***Note.*** Individuals choosing ‘I don’t know’ has been categorized as normal hearing. The version presented to German speaking participants is presented in Table A2.

**Table A2**

*Self-assessment of individual’ hearing impairment in German language*

| **Question** | | | | | | |  |
| --- | --- | --- | --- | --- | --- | --- | --- |
| Haben Sie das Gefühl, dass Sie einen Hörverlust haben? | | | | | | |  |
| *(Auch wenn dies nicht genau auf Ihre Erfahrung zutrifft, sollten Sie sich überlegen, welche dieser Angaben Ihr Gehör ohne Hörgeräte am besten beschreibt.)* | | | | | | |  |
|  |  |  |  |  |  |  |  |
| **Options** | | | | | | |  |
| □ Nein, ich habe nicht das Gefühl einen Hörverlust zu haben. | | | | | | |  |
| □ Ja, ich habe das Gefühl, leichtgradig schwerhörig zu sein (Hörverlust von 20–40 dB): Wenn ich mit einer Person in einem ruhigen Raum spreche, kann ich normalerweise ein Gespräch verstehen. In lauten Situationen (z. B. in einer Kneipe) und bei Gruppengesprächen habe ich manchmal Probleme, Sprache zu verstehen. | | | | | | |  |
| □ Ja, ich habe das Gefühl, mittelgradig schwerhörig zu sein (Hörverlust von 41–60 dB): Ich habe Schwierigkeiten, Sprache zu verstehen, und muss häufig andere bitten, das Gesagte zu wiederholen, sowohl im persönlichen Gespräch als auch am Telefon. In lauten Situationen fällt es mir schwer, Gesprächen zu folgen. Ich muss den Fernseher und das Radio lauter stellen, um sie deutlich zu hören. | | | | | | |  |
| □ Ja, ich habe das Gefühl, hochgradig schwerhörig zu sein (Hörverlust von 61–80 dB): Ich kann Sprache ohne Hörgeräte kaum verstehen und benutze Lippenlesen/Gebärdensprache zur Unterstützung der Kommunikation. | | | | | | |  |
| □ Ja, ich habe das Gefühl, an Taubheit grenzend schwerhörig zu sein (Hörverlust von 81–95 dB): Gewöhnliche Gespräche sind unverständlich. Ich kann geschriebene Wörter nicht hören und muss mich auf das Lippenlesen und/oder Gebärdensprache verlassen, um mich zu verständigen. | | | | | | |  |
| □ Ich weiß nicht. | | | | | | |  |

***Note.*** Individuals choosing ‘Ich weiß nicht’ has been categorized as normal hearing. The version presented to English speaking participants is presented in Table A1.

**Table A3**

*Musicians average sound level exposure per instrument*

| Instrument Group | Instruments | sound level estimates (dB A) | | | | | | | |
| --- | --- | --- | --- | --- | --- | --- | --- | --- | --- |
|  |  | Schmidt et al., 2011^a^ | | O’Brien et al., 2013^b^ | Phillips & Mace, 2008^c^ | Laitinen et al., 2003^d^ | | Overall | |
|  |  | *IP* | *GP* | *IP* | *IP* | *IP* | *GP* | *IP* | *GP* |
| Violins | 1^st^ Violin | 89^e^ | 86 | 87.5^e^ | 87^e^ | 86^e^ | 88.3^e^ | 87.4 | 87.2 |
|  | 2^nd^ Violin | 89^e^ | 86.2 | 87.5^e^ | 87^e^ | 86^e^ | 88.3^e^ | 87.4 | 87.3 |
| Violas/Cellos | Violoncello | - | 81.8 | 80 | 87^e^ | 88 | 89.8 | 85.0 | 85.8 |
|  | Viola | 90.5 | 87.7 | 85.5 | 87^e^ | 86 | 90.3 | 87.3 | 89 |
| Woodwinds | Flute/ Piccolo | 101.2 | 90.4 | 94 | 90.4^e^ | 96 | 90.7^e^ | 95.4 | 90.6 |
|  | Oboe | - | 86.7 | 85 | 90.4^e^ | 91^e^ | 90.7^e^ | 88.8 | 88.7 |
|  | Bassoon | 88.4 | 88 | 88 | 90.4^e^ | 91^e^ | 90.7^e^ | 89.5 | 89.4 |
|  | Clarinet | - | 88.6 | 95 | 90.4^e^ | 91^e^ | 90.7^e^ | 92.1 | 89.7 |
| Brass | Trombone | 97.6 | 89.7 | 96 | 95.2^e^ | 95 | 93^e^ | 96.0 | 91.4 |
|  | Trumpet | 90.9 | 92 | 95.5 | 95.2^e^ | 97 | 92.3 | 94.7 | 92.2 |
|  | Horn | - | - | 93.5 | 95.2^e^ | 95 | 93^e^ | 94.6 | 93 |
|  | Tuba | - | 87.8 | 93.5 | 95.2^e^ | 95 | 93^e^ | 94.6 | 90.4 |
| Percussion/  Bass | Contrabass | 75.6 | 82.7 | 75 | 90.1^e^ | 79 | 87 | 79.9 | 84.9 |
|  | Kettledrum/ Timbals/ Side drums | - | 87.2 | 97.5 | 90.1^e^ | 99 | 91.3^e^ | 95.5 | 89.3 |
| Others | Harp | - | - | 83 | - | 89 | 87 | 86.0 | 87 |
|  | Celeste | - | 90 | - | - | - | - | 90.0 | 90 |
|  |  |  |  |  |  |  |  |  |  |

***Note.*** Hours of noise exposure per week was estimated by aggregating musicians' reported average playing durations across various settings - namely, individual practice and group performances (IP = individual practice; GP = group practice). While it's reasonable to assume consistent playing habits throughout the year, these averages were projected over a standard 44-week year (equivalent to 1,780 working hours instead of 2080 hours annually, as is typical in Germany) to calculate annual noise exposure. Sound exposure levels differ notably across instruments, musicians' repertoires, and between solo practice and group performances (e.g., Rodrigues et al., 2014). To accommodate these variances, the metric derived sound level estimates from average decibel level data for various instruments and ensembles, playing in different acoustical settings and repertoires, as sourced from existing literature (Laitinen et al., 2003; O'Brien et al., 2013; Phillips & Mace, 2008; Schmidt et al., 2011). Furthermore, sound level estimates that are experienced by musicians were calculated by considering individual practice sessions and group performances separately.
^a^ Estimates reflect measurements from two orchestras during group rehearsals and actual concerts featuring varied repertoires, including Niels W. Gade's "Ossian Overture" and Dmitri Shostakovich's "Violin Concerto No. 1." Average measurements from both the left and right ears are reported.
^b^ Measurements of a daily practice session of duration (2.1 h) according to the average from both the left and right ears in a 4.8m x 4m room. Fortissimo passages, which can increase levels by up to 8 dB, are not included in these measurements. ^c^ Average estimates per instrument group were acquired by measuring students practicing in approx. 3m x 3.66m. ^d^ Average sound level for performances and orchestra rehearsals of one orchestra playing ‘Die Walküre’, ‘Insect Life’, ‘Don Giocanni’, and ‘Swan Lake’. ^e^ Measurements are averaged over instrument groups.

**Table A4**

*Musicians average sound level exposure per instrument group*

| **Instrument  Group** | **Instruments in group** | **sound level  exposure estimate (dB A)** | |
| --- | --- | --- | --- |
|  |  | **individual  practice** | **rehearsals / concerts** |
| Violins | 1st Violin, 2nd Violin | 87.4 | 87.2 |
| Violas/Cellos | Violoncello, Viola | 86.1 | 87.4 |
| Woodwinds | Flute/Piccolo, Oboe, Bassoon, Clarinet | 91.4 | 89.6 |
| Brass | Trombone, Trumpet, Horn, Tuba | 94.9 | 91.7 |
| Percussion/Bass | Contrabass, Kettledrum/Timbals | 87.7 | 87.1 |
| Others | Harp, Celeste | 88 | 88.5 |

***Note.***  Estimates are based on averages per instrument group from several sound level measurements provided by the literature as reported in Table A3 (see Supp. Mat.; Laitinen et al., 2003; Phillips & Mace, 2008; O’Brien et al., 2013; Schmidt et al., 2011) calculated for individual practice and rehearsals / orchestral concert performances separately. Notably, estimated exposure levels for individual practice sessions are reported to be higher, compared to group performances. The main contributing factor to this higher exposure is the continuous play during individual practice sessions compared to group rehearsals (e.g., see Schmidt et al., 2011). The instrument classification was from Russo et al. (2013).

**Table A5**

*Types of hearing protection: Average attenuation levels and usage per group*

| **Hearing protection** |  |
| --- | --- |
|  | ***attenuation dB*** ^d^ |
| DIY [e.g., cotton wool] ^a^ | 7 |
| Standard ear plugs ^b^ | 31 |
| Plastic ear plugs ^b^ | 26 |
| Balanced sound-reducing plugs ^b^ | 16 |
| Customised ear plugs ^c^ | 22 |
| None | 0 |

***Note.***  To establish the probability estimate for the attenuation within the unit of noise exposure, reported frequency of hearing protection usage was converted into probability estimates: "Never" equals 0% probability of wearing hearing protection, "rarely" = 25%, "sometimes" = 50%, "mostly" = 75%, and "always" = 100%. Attenuation values (A) of the respective hearing protection devices (ear plugs) were estimated based on the hearing protection guide reported in the literature; ^a^ Safety Research Corporation of America (2020). ^b^ Guest et al. (2018). ^c^ Kusy & Châtillon (2012) ^d^ Overall estimates are provided.

**Table A6**

*Types of hearing protection: Average attenuation levels and usage per group*

| **Hearing protection** |  | | **N (prop)^d^** | | |
| --- | --- | --- | --- | --- | --- |
|  | ***attenuation dB*** | ***amateurs*** | | ***professional*** |  |
| DIY [e.g., cotton wool] ^a^ | 7 | 0 | | 3 (.015) |  |
| Standard ear plugs ^b^ | 31 | 5 (.025) | | 5 (.025) |  |
| Plastic ear plugs ^b^ | 26 | 0 | | 4 (.02) |  |
| Balanced sound-reducing plugs ^b^ | 16 | 3 (.015) | | 5 (.025) |  |
| Customised ear plugs ^c^ | 22 | 15 (.075) | | 89 (.445) |  |
| None | 0 | 177 (.885) | | 94 (.47) |  |

***Note.***  The attenuation depends on several factors, including the frequency of the signal; here overall measures are provided. ^a^ Safety Research Corporation of America (2020). ^b^ Guest et al. (2018). ^c^ Kusy & Châtillon (2012). ^d^ N (responses) for both groups of musicians after the matching procedure. Proportions are provided in brackets.

**Table A7**

*Results from CFA for items indicating music-related hearing problems*

| **Item** | | | | | | Mean* (SD) | | | Corrected Item-Total  Correlation | Cronbach’s α if Item Deleted | Factor Loadings  (lambda) |
| --- | --- | --- | --- | --- | --- | --- | --- | --- | --- | --- | --- |
| **No** | When making music and listening to music... | | | | | |  | |  |  |  |
| 1 | ...I have the impression that the sound is washed   out/ blurred. | | | | | | 4.6 (.74) | | .62 | .80 | .61 |
| 2 | ...I cannot hear well what I am playing. | | | | | | 4.2 (1.02) | | .63 | .80 | .63 |
| 3 | ...I cannot hear well what other orchestra members   are playing. | | | | | | 4.0 (.94) | | .77 | .76 | .8 |
| 4 | ...I have problems perceiving intonation or pitch  differences clearly. | | | | | | 4.3 (.86) | | .70 | .78 | .71 |
| 5 | ...I have difficulty recognising other instruments. | | | | | | 4.5 (.69) | | .66 | .79 | .65 |
| 6 | ...the music is often too soft or too loud. | | | | | | 4.0 (1.00) | | .57 | .81 | .57 |
|  |  |  |  |  |  |  | |  |  |  |  |
| **Model Fit Indices** | | | | | | | | | | Value | References |
| Robust Comparative Fit Index | | | | | | | | | | .994 | > .95 |
| Robust Tucker-Lewis Index | | | | | | | | | | .990 | > .95 |
| Robust RMSEA | | | | | | | | | | .050 | < .06 |
| SRMR | | | | | | | | | | .045 | < .08 |
| R^2^ | | | | | | | | | | .44 |  |
| Cronbach’s α | | | | | | | | | | .82 |  |

***Note.*** N = 575. Raw Cronbach's α = .82, 95% CI[0.8, 0.84]. The kurtosis values are within acceptable ranges. Adequacy of the analysis was assessed by the Kaiser-Meyer-Olkin index (.84) and Bartlett’s test of sphericity (χ²(21) = 1261, p < .001). Participants rated items on a 5-Point-Likert-Scale, ranging from 1 = always to 5 = never. *Low scores represent participants consistently providing the most extreme responses on all the assessed items, signifying intense experiences with hearing issues in the context of music.

**Table A8**

*Results from CFA for items indicating hearing health awareness (HHA)*

| **Item** | | | | | | Mean (SD) | | | Corrected Item-Total  Correlation | Cronbach’s α if Item Deleted | Factor Loadings  (lambda) |
| --- | --- | --- | --- | --- | --- | --- | --- | --- | --- | --- | --- |
| **No** |  | | | | | |  | |  |  |  |
| 1 | Hearing health is extremely important to me. | | | | | | 1.3 (.5) | | .23 | .78 | .18 |
| 2 | I have my hearing checked regularly. | | | | | | 2.62 (1.1) | | .40 | .77 | .34 |
| 3 | The sound levels in the orchestra are so high at times that I should wear hearing protection. | | | | | | 2.1 (1.1) | | .80 | .69 | .86 |
| 4 | Do you perceive the sound level exposure in the orchestra profession as a risk to your hearing health? | | | | | | 2.19 (1.5) | | .78 | .69 | .81 |
| 5 | How do you feel about the sound level exposure during the individual training periods? | | | | | | 3.43 (.7) | | .30 | .70 | .29 |
| 6 | How do you feel about the sound level exposure during the rehearsals? | | | | | | 2.3 (.8) | | .74 | .68 | .76 |
| 7 | How do you feel about the sound level exposure during concerts? | | | | | | 2.2 (.8) | | .66 | .69 | .66 |
|  |  |  |  |  |  |  | |  |  |  |  |
| **Model Fit Indices** | | | | | | | | | | Value | References |
| Robust Comparative Fit Index | | | | | | | | | | .973 | > .95 |
| Robust Tucker-Lewis Index | | | | | | | | | | .959 | > .95 |
| Robust RMSEA | | | | | | | | | | .081^*^ | < .06 |
| SRMR | | | | | | | | | | .062 | < .08 |
| R^2^ | | | | | | | | | | .37 |  |
| Cronbach’s α | | | | | | | | | | .76 |  |

***Note****.* N = 553. Raw Cronbach's α = .76, 95% CI[.73, .79]. The kurtosis values are within acceptable ranges. Adequacy of the analysis was assessed by the Kaiser-Meyer-Olkin index (.79) and Bartlett’s test of sphericity (χ²(21) = 454, p < .001). Participants rated items on a 4-Point-Likert-Scale, ranging from 1 = “true/applies” to 4 = “not true/does not apply”. *RMSEA is .081 90% CI [.061, .101] with RMSEA values less than .05 indicate a close fit, values up to .08 indicate a reasonable error of approximation, and values greater than .10 suggest a poor fit. This model still falls into the category of a reasonable fit. All items were used to compute the final factor score, as excluding any items did not noticeably enhance the reliability. The final scores were transformed to span from 0 to 10, with higher values indicating a higher hearing health awareness.

**Table A9**

*Results from CFA for items indicating hearing-aid disapproval (HAD)*

| Item | | | | | | Mean (SD) | | Corrected Item-Total  Correlation | Cronbach’s α if Item Deleted | Factor Loadings  (lambda) |
| --- | --- | --- | --- | --- | --- | --- | --- | --- | --- | --- |
| **No** |  | | | | |  | |  |  |  |
| 1 | It irritates me, when colleagues with hearing impairments wear hearing aids. | | | | | 3.59 (.71) | | .62 | .79 | .48 |
| 2 | If there were invisible hearing aids, I would have less problems wearing one. | | | | | 2.96 (1.14) | | .69 | .78 | .53 |
| 3 | If I wore a hearing aid, I would be afraid of losing respect among my colleagues. | | | | | 3.06 (1.02) | | .84 | .70 | .87 |
| 4 | Wearing a hearing aid would leave a bad image among the audience. | | | | | 3.08 (.97) | | .83 | .70 | .85 |
| 5 | Making music professionally in an orchestra and wearing hearing aids are mutually exclusive. | | | | | 3.25 (.9) | | .71 | .76 | .577 |
|  |  |  |  |  |  |  |  |  |  |  |
| **Model Fit Indices** | | | | | | | | | Value | References |
| Robust Comparative Fit Index | | | | | | | | | .996 | > .95 |
| Robust Tucker-Lewis Index | | | | | | | | | .991 | > .95 |
| Robust RMSEA | | | | | | | | | .058 | < .06 |
| SRMR | | | | | | | | | .037 | < .08 |
| R^2^ | | | | | | | | | .463 |  |
| Cronbach’s α | | | | | | | | | .79 |  |

***Note.*** N = 553. Raw Cronbach's α = 0.79, 95% CI [.76, .82]. All five items show left-skewed distributions, with varying degrees of skewness. Additionally, all items have leptokurtic distributions. CFA was thus conducted after transforming the date (cubric transformation). Adequacy of the analysis was assessed by the Kaiser-Meyer-Olkin index (.78) and Bartlett’s test of sphericity (χ²(10) = 854, p < .001). Participants rated items on a 4-Point-Likert-Scale, ranging from 1 = “true/applies” to 4 = “not true/does not apply”. The scores derived from this model were transformed to range from 0 to 10, where higher values indicate a greater disapproval towards hearing aids.

**Figures**

**Figure A1**

**
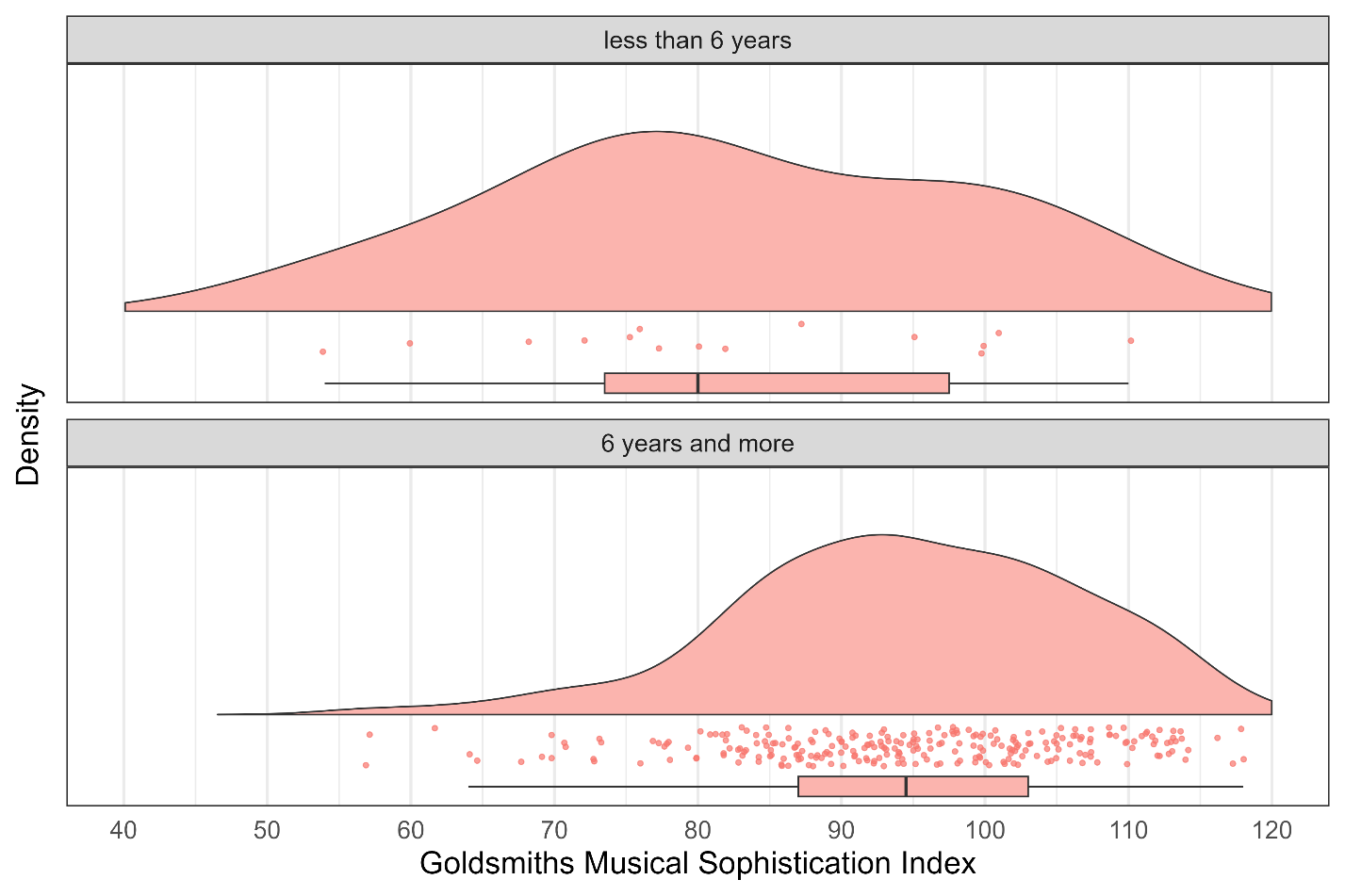

Fig. A1.**  Density plots, along with boxplots and individual data points (pale red dots), illustrate the distribution of Goldsmiths Musical Sophistication Index (GMSI; Müllensiefen et al, 2014) scores. These plots compare two groups within the amateur musician sample: participants with <6 years of daily practice (top panel) and the main sample after exclusion of the <6 years practice group (bottom panel). Higher GMSI scores (range: 18-126) indicate higher musical sophistication. The top panel median GMSI (M = 80) is comparable to the general population (M = 77-82; see Müllensiefen et al., 2014). These participants have been excluded, following the criteria by Zhang & Schubert (2019). The main sample median GMSI (bottom panel) is 95 (SD = 12.01). Results shown are before matching.

**Figure A2**


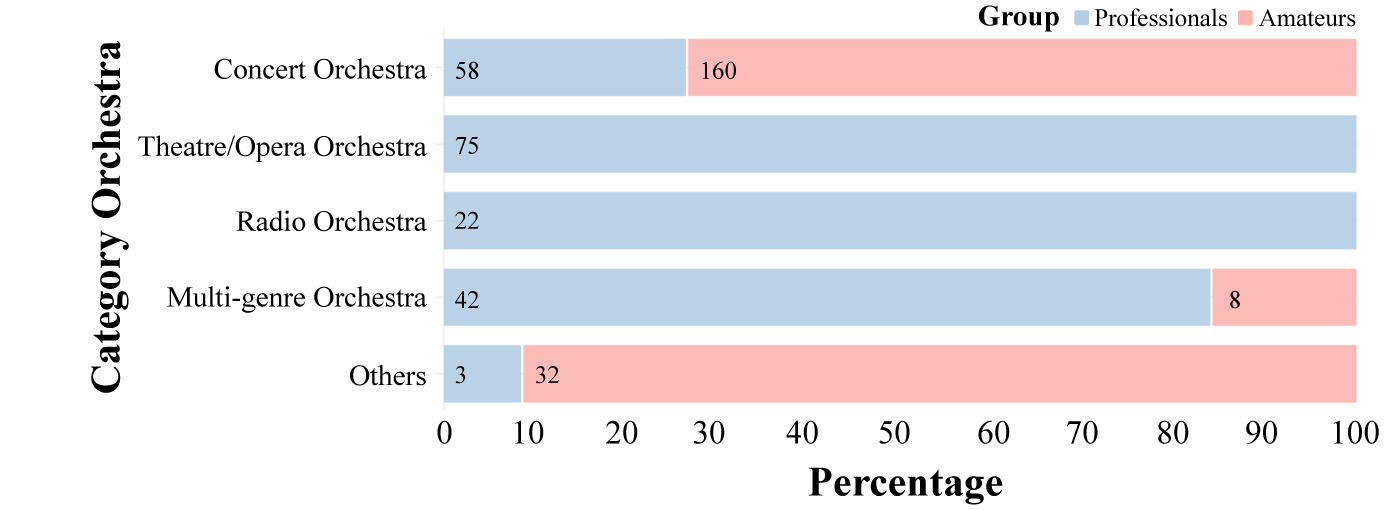


**Fig. A2**. Distribution of primarily played orchestral category/repertoire among respondents, shown post-matching procedure. Light blue bars represent professional musicians, and pale bars represent amateurs. Numbers inside each bar indicate the count within each respective category.

**Figure A3**

**
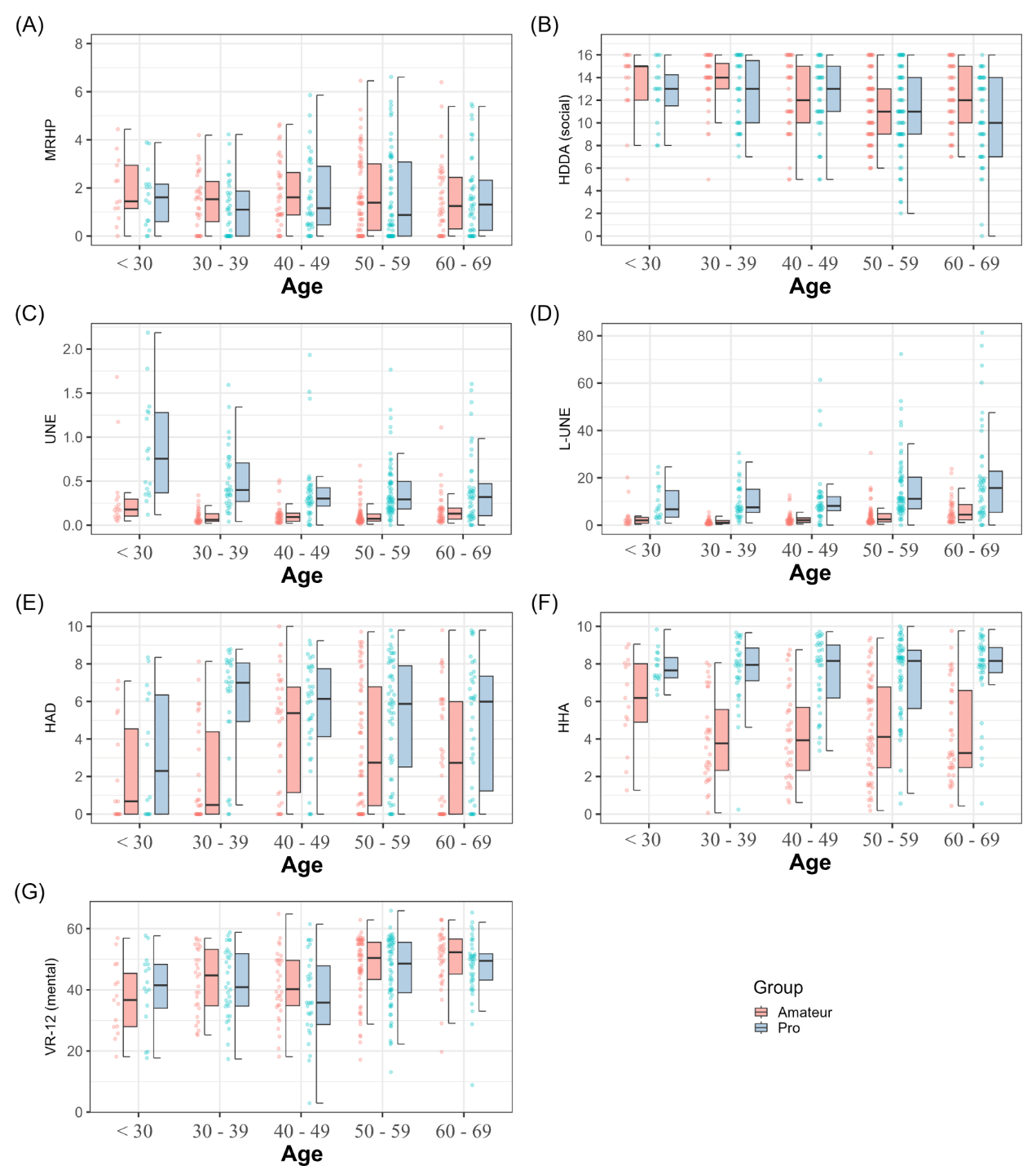
**

**Fig. A3.** This multi-figure depicts the distribution of hearing-related measures and music exposure across age groups in the matched sample of amateur and professional musicians (N = 400). Boxplots show grouped distributions, with dots representing individual responses (amateurs: pale dots, professionals: light blue dots). Panels depict: (A) Music-Related Hearing Problems (MRHP), (B) Hearing-Dependent Daily Activities Subscale for social interactions (HDDA), (C) Unit of Noise Exposure (UNE), (D) Lifetime UNE (L-UNE), (E) Hearing Aid Disapproval (HAD), (F) Hearing Health Awareness (HHA), (G) Veterans Health-related quality of life (VR-12) subscale for mental health.

**Figure A4**

**
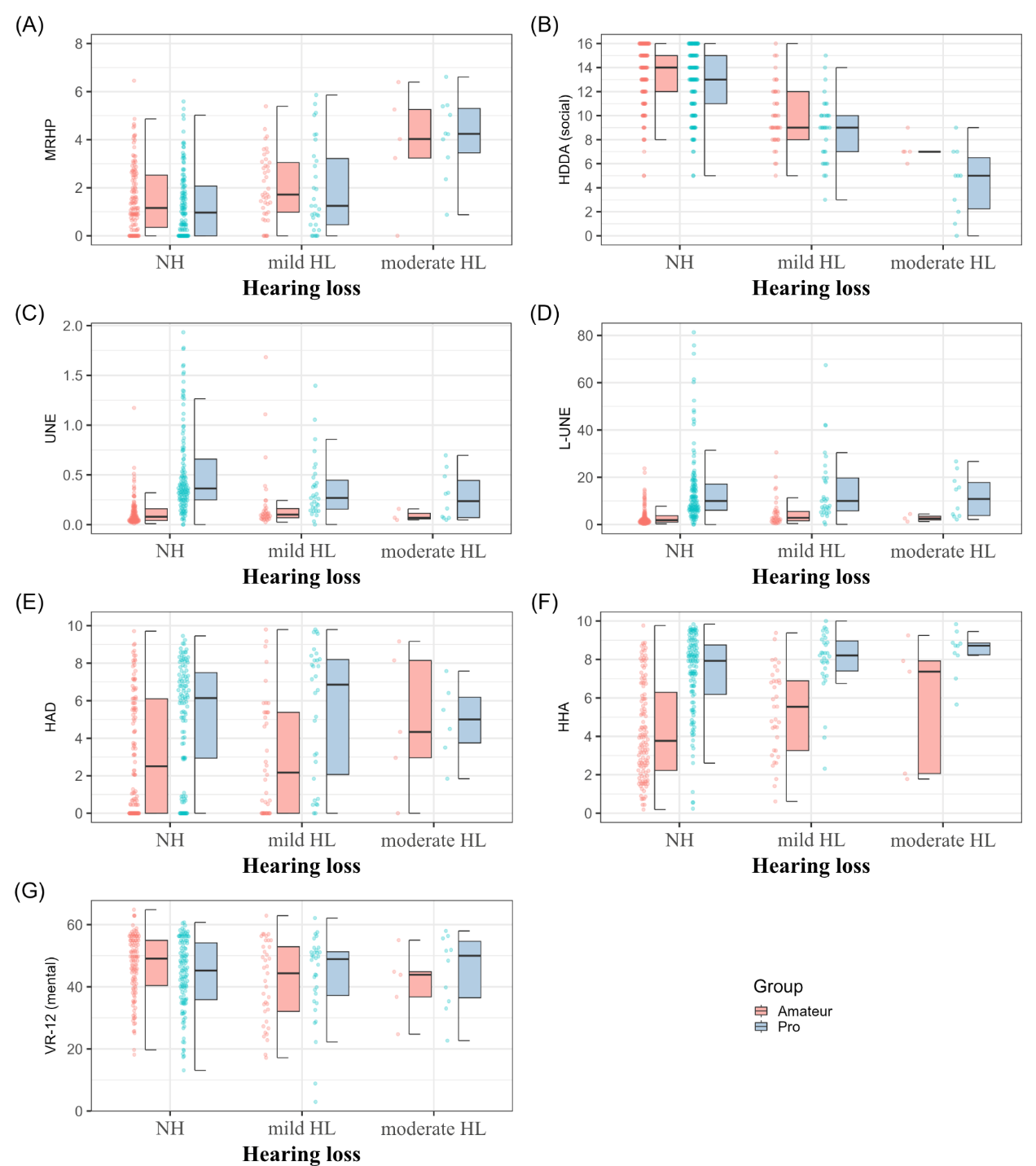
**

**Fig. A4.** This multi-figure depicts the distribution of hearing-related measures and music exposure across hearing loss groups in the matched sample of amateur and professional musicians (N = 400). Boxplots show grouped distributions, with dots representing individual responses (amateurs: pale dots, professionals: light blue dots). Panels depict: (A) Music-Related Hearing Problems (MRHP), (B) Hearing-Dependent Daily Activities Scale (HDDA), (C) Unit of Noise Exposure (UNE), (D) Lifetime UNE (L-UNE), (E) Hearing Aid Disapproval (HAD), (F) Hearing Health Awareness (HHA), (G) Veterans Health-related quality of life (VR-12). (HL

**Literature**

Guest, H., Dewey, R. S., Plack, C. J., Couth, S., Prendergast, G., Bakay, W., & Hall, D. A. (2018). The Noise Exposure Structured Interview (NESI): An Instrument for the Comprehensive Estimation of Lifetime Noise Exposure. *Trends in Hearing*, 22. <https://doi.org/10.1177/2331216518803213>

Kusy, A., & Châtillon, J. (2012). Real-world attenuation of custom-moulded earplugs: Results from industrial in situ F-MIRE measurements. *Applied Acoustics, 73*(6–7), 639–647. <https://doi.org/10.1016/j.apacoust.2012.02.001>

Laitinen, H. M., Toppila, E. M., Olkinuora, P. S., & Kuisma, K. (2003). Sound exposure among the Finnish National Opera personnel. *Applied Occupational and Environmental Hygiene, 18*(3), 177–182. <https://doi.org/10.1080/10473220301356>

Müllensiefen, D., Gingras, B., Musil, J., & Stewart, L. (2014). The musicality of non-musicians: An index for assessing musical sophistication in the general population. *PLoS ONE, 9*(2). <https://doi.org/10.1371/journal.pone.0089642>

O’Brien, I., Driscoll, T., & Ackermann, B. (2013). Sound exposure of professional orchestral musicians during solitary practice. *The Journal of the Acoustical Society of America, 134*(4), 2748–2754. <https://doi.org/10.1121/1.4820900>

Phillips, S. L., & Mace, S. (2008). Sound level measurements in music practice rooms. *Music Performance Research, 2*(1993), 36–47.

Rodrigues, M. A., Freitas, M. A., Neves, M. P., & Silva, M. V. (2014). Evaluation of the noise exposure of symphonic orchestra musicians. *Noise & health, 16*(68), 40–46. <https://doi.org/10.4103/1463-1741.127854>

Russo, F. A., Behar, A., Chasin, M., & Mosher, S. (2013). Noise exposure and hearing loss in classical orchestra musicians. *International Journal of Industrial Ergonomics, 43*(6), 474–478. <https://doi.org/10.1016/j.ergon.2012.11.001>

Safety Research Corporation of America (2020). *Noise and hearing protection fact sheet*. Retrieved July 5, 2023, from <https://www.srca.net/Blog/NoiseAndHearingProtectionFactSheet.aspx#:~:text=Why%20Can%E2%80%99t%20I,as%20adequate%20protection>

Schmidt, J. H., Pedersen, E. R., Juhl, P. M., Christensen-Dalsgaard, J., Andersen, T. D., Poulsen, T., & Bælum, J. (2011). *Sound exposure of symphony orchestra musicians. Annals of Occupational Hygiene, 55*(8), 893–905. <https://doi.org/10.1093/annhyg/mer055>

Zhang, J. D., & Schubert, E. (2019). A Single Item Measure for Identifying Musician and Nonmusician Categories Based on Measures of Musical Sophistication. *Music Perception, 36*(5), 457–467. <https://doi.org/10.1525/mp.2019.36.5.457>
